# Comprehensive analysis of the Co-structures of dipeptidyl peptidase IV and its inhibitor

**DOI:** 10.1186/s12900-016-0062-8

**Published:** 2016-08-05

**Authors:** Hiroyuki Nojima, Kazuhiko Kanou, Genki Terashi, Mayuko Takeda-Shitaka, Gaku Inoue, Koichiro Atsuda, Chihiro Itoh, Chie Iguchi, Hajime Matsubara

**Affiliations:** 1 School of Pharmacy, Kitasato University, 5-9-1 Shirokane, Minato-ku Tokyo, 108-8641 Japan; 2 Present address: Infectious Disease Surveillance Center, National Institute of Infectious Diseases, 1-23-1 Toyama, Shinjuku-ku Tokyo, 162-8640 Japan

**Keywords:** Dipeptidyl peptidase IV, DPP-4 inhibitor, Inhibitory activity, Cocrystal structure, Water molecule, In silico screening

## Abstract

**Background:**

We comprehensively analyzed X-ray cocrystal structures of dipeptidyl peptidase IV (DPP-4) and its inhibitor to clarify whether DPP-4 alters its general or partial structure according to the inhibitor used and whether DPP-4 has a common rule for inhibitor binding.

**Results:**

All the main and side chains in the inhibitor binding area were minimally altered, except for a few side chains, despite binding to inhibitors of various shapes. Some residues (Arg125, Glu205, Glu206, Tyr662 and Asn710) in the area had binding modes to fix a specific atom of inhibitor to a particular spatial position in DPP-4. We found two specific water molecules that were common to 92 DPP-4 structures. The two water molecules were close to many inhibitors, and seemed to play two roles: maintaining the orientation of the Glu205 and Glu206 side chains through a network via the water molecules, and arranging the inhibitor appropriately at the S2 subsite.

**Conclusions:**

Our study based on high-quality resources may provide a necessary minimum consensus to help in the discovery of a novel DPP-4 inhibitor that is commercially useful.

**Electronic supplementary material:**

The online version of this article (doi:10.1186/s12900-016-0062-8) contains supplementary material, which is available to authorized users.

## Background

Incretin is an endogenous gut hormone that is useful in treating patients with type 2 diabetes [1]. Incretin is secreted from the digestive tract with dietary intake [2] and acts on pancreatic β-cells to stimulate insulin secretion [1, 3]. Sulfonylurea, a traditional hypoglycemic drug, promotes insulin secretion regardless of the blood glucose level; because of this, it risks eliciting serious hypoglycemia. In contrast, an antidiabetic drug that uses incretin as a mediator is expected to reduce the risk of hypoglycemia because stimulation of insulin secretion by incretin depends on the blood glucose level [4].

Glucagon-like peptide-1 (GLP-1) is an incretin with strong insulin secretion effect [5]. The active form of GLP-1 comprises 30 amino acids [GLP-1-(7–36)NH_2_ or GLP-1-(7–37)] [6], but it has a short half-life of only 2 min because two residues (His-Ala) on the N-terminus of the active form are removed by dipeptidyl peptidase IV (DPP-4) [7]. Currently, two different types of drugs are in clinical use to target GLP-1. The first type is a GLP-1 analog, which has a longer half-life than active endogenous GLP-1; examples of this type include liraglutide (half-life = 13 h) [8], exenatide (half-life = 1.3–1.6 h) [9] and lixisenatide (half-life = approximately 2 h) [10]. The second type is a DPP-4 inhibitor, which prolongs the half-life of active endogenous GLP-1 by inhibiting DPP-4.

DPP-4 has strong protease activity against polypeptides that have an alanine or proline as a second N-terminal residue [11]. DPP-4 inhibitor development began with dipeptide structures that included alanine or proline as the base. Currently, nine DPP-4 inhibitors are marketed in many countries: sitagliptin [2], vildagliptin [12, 13], alogliptin [14, 15], linagliptin [16], anagliptin [17, 18], teneligliptin [19], saxagliptin [20], trelagliptin [15, 21] and omarigliptin [22]. However, these nine drugs have different structures, with the exception of vildagliptin and saxagliptin, or alogliptin and trelagliptin [23–25] (Table 1).

**Table 1:** Structural similarity of commercial DPP-4 inhibitors (Tanimoto coefficient^a^)

	Sitagliptin	Vildagliptin	Alogliptin	Linagliptin	Teneligliptin	Anagliptin	Saxagliptin	Trelagliptin	Omarigliptin
Sitagliptin (IC_50_^b^: 18 nM)	-								
Vildagliptin (IC_50_: 3.5 nM)	0.351	-							
Alogliptin (IC_50_: 7 nM)	0.325	0.424	-						
Linagliptin (IC_50_: 1 nM)	0.286	0.239	0.364	-					
Teneligliptin (IC_50_: 0.37 nM)	0.261	0.300	0.341	0.413	-				
Anagliptin (IC_50_: 3.8 nM)	0.244	0.429	0.233	0.286	0.349	-			
Saxagliptin (Ki: 0.6 nM)	0.378	**0.800**	0.371	0.289	0.233	0.308	-		
Trelagliptin (IC_50_: 4 nM)	0.350	0.412	**0.962**	0.356	0.366	0.227	0.361	-	
Omarigliptin (IC_50_: 1.6 nM, Ki: 0.8 nM)	0.410	0.324	0.368	0.292	0.326	0.146	0.250	0.395	-

^a^ Tanimoto coefficients were calculated by chemical structure comparison using the build-up algorithm [23]. They range from 0 to 1. When 0.8 or higher, two structures are evaluated as similar (bold)

^b^ IC_50_ and Ki were quoted from the Web Server “The Binding Database”, http://www.bindingdb.org/bind/ [24] or the Web Server “PDB bind”, http://www.pdbbind.org.cn/ [25]

**Fig. 1:**
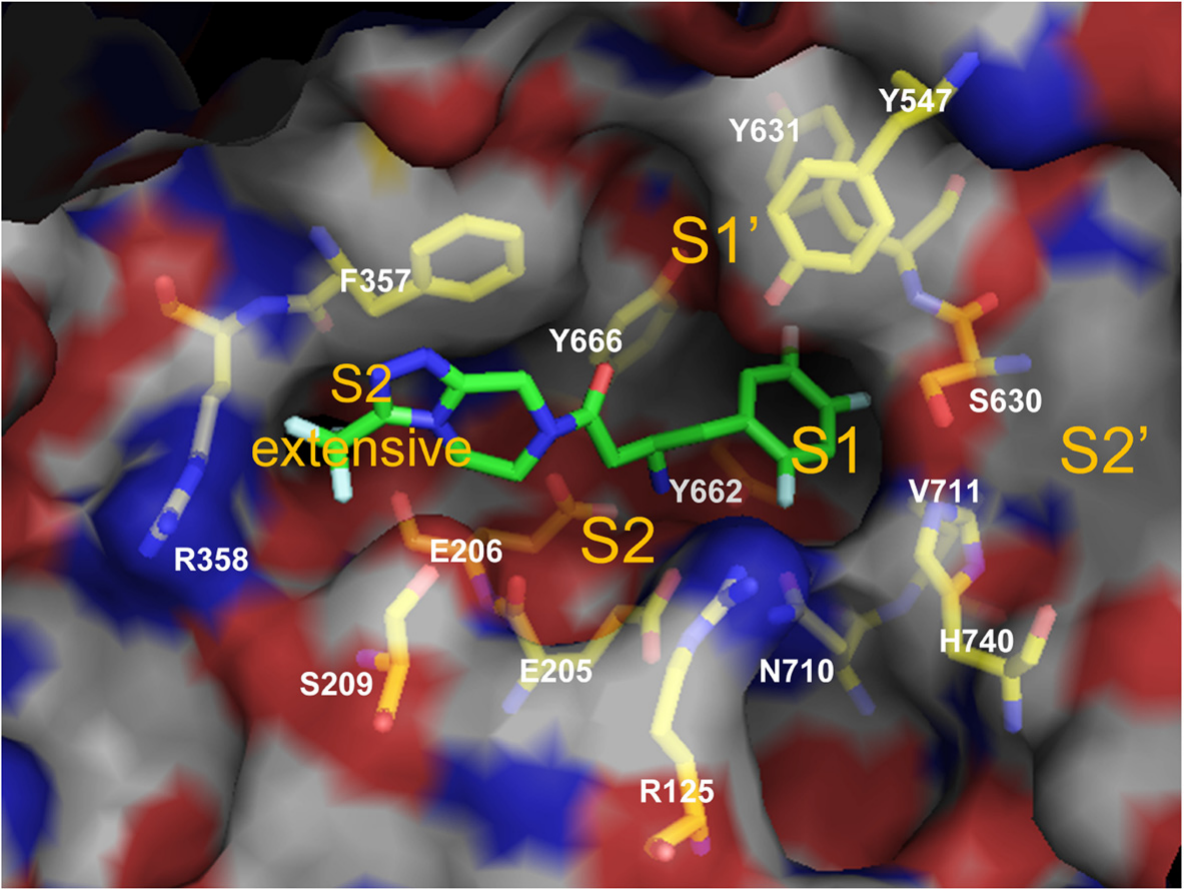
Inhibitor binding area of DPP-4. Representative image from the cocrystal structure of sitagliptin and DPP-4 (PDB ID: 1X70). The carbon skeleton of sitagliptin is represented by green stick. The carbon skeleton of 14 residues is labeled and represented by yellow stick. Val656 and Trp659 are positioned on the opposite side of view in this Figure; thus, they are not shown. O, N, and halogen atoms are labeled in red, blue and light blue sticks, respectively. The subsites, which are directly involved with binding to inhibitors (S1, S1′, S2, S2′ and S2 extensive), are labeled in orange

An explanation for why DPP-4 can accept inhibitors of various shapes is that DPP-4 has a large cavity (diameter ≥20 Å) [26], and these inhibitors may be allowed to approach the active center of DPP-4. In addition, DPP-4 has multiple binding subsites known as the S1, S2, S1’, S2’ and S2 extensive subsites (Fig. 1) [13]. Of the commercial drugs, vildagliptin and saxagliptin bind to the S1 and S2 subsites, alogliptin, linagliptin and possibly trelagliptin bind to the S1’ and/or S2’ subsites in addition to the S1 and S2 subsites, while sitagliptin, anagliptin, teneligliptin and omarigliptin bind to the S1, S2 and S2 extensive subsites. The commercial drugs efficiently match the energy in these subsites and, in this manner, probably attain high DPP-4 inhibitory activity.

It is not known if there are other causes for DPP-4 binding to inhibitors of various shapes. For example, does DPP-4 alter its general or partial structure according to the inhibitor used? Or, does DPP-4 have a common rule for inhibitor binding? To answer these questions, we comprehensively analyzed X-ray cocrystal structures of DPP-4 and its inhibitor. All the main and side chains in the inhibitor binding area were minimally altered, except for some side chains, despite binding to inhibitors of various shapes. Some residues in the area had binding modes to fix a specific atom of inhibitor to a particular spatial position in DPP-4. We found two specific water molecules that were common to many DPP-4 structures. The two water molecules were close to many inhibitors, and seemed to be related to inhibitor binding. This information may provide a necessary minimum consensus to help in the discovery of a novel DPP-4 inhibitor that is commercially useful.

## Methods

### Data collection

We collected X-ray cocrystal structures of human DPP-4 and its inhibitor that were registered with the Protein Data Bank (PDB) [27] until 2015. Sixty-eight PDB codes that had a resolution of less than 3 Å were used (Additional file 1: Figure S1). Most of the PDBs had a crystallization temperature that ranged from 277 K to 298 K and an X-ray diffraction-measured temperature range from 90 K to 120 K. However, the X-ray diffraction of only five PDBs (PDB ID: 2AJL, 2I03, 2I78, 2OLE and 3EIO) was measured at a high temperature (in the range of 200 K to 298 K) (Additional file 2: Table S1).

One, two or four DPP-4 molecules are included per one PDB code. The DPP-4 molecule that had an inhibitor bound to the DPP-4 active center and that had less than six disordered residues was selected from each PDB code. We defined the coordinates of one DPP-4 molecule (724 residues: residue 41–764) with one inhibitor on the active center and water O atoms within 4 Å from the DPP-4 molecule as one unit (we will discuss the distance between heavy atoms). Ultimately, there were 147 inhibitor-bound units identified from the 68 PDBs (i.e., 68 kinds of inhibitors). To compare and evaluate the inhibitor-bound units, we also collected X-ray crystal structures of inhibitor-free human DPP-4 that had a resolution of less than 3 Å. Eight inhibitor-free units were identified from four PDB codes (PDB ID: 1J2E, 1NU6, 1PFQ and 1TK3) (Additional file 2: Table S1). These units were used for the procedure discussed below.

### Defining the DPP-4 inhibitor binding area

Generally, some kinds of interactions (e.g., hydrogen bond, electrostatic interaction, hydrophobic interaction and π–π stacking effect) are considered to occur between two heavy atoms that are close to each other (less than c.a. 4–5 Å). In DPP-4, 13 residues (Arg125, Glu205, Glu206, Phe357, Tyr547, Ser630, Tyr631, Val656, Tyr662, Tyr666, Asn710, Val711 and His740) were close to (<4 Å) at least 48 out of the 68 inhibitors (>70 %) and three residues (Ser209, Arg358 and Trp659) were close to (<4 Å) at least 21 out of the 68 inhibitors (>30 %). We defined the above 16 residues as the DPP-4 inhibitor binding area (Fig. 1).

### Cα atom variation between units

We calculated Cα atom variation between units as follows:

- [Step 1] From the above-mentioned units, two units were selected.
- [Step 2] The two units were superimposed so that the root mean square deviation (RMSD) targeting Cα atoms of the DPP-4 molecule (residue 41–764) would be minimized. The RMSD is generally defined by the following Eq. (1):

1 $$ \mathrm{RMSD}=\sqrt{\frac{1}{\mathrm{N}}{\displaystyle \sum_i^{\mathrm{N}}{x}_i^2}} $$

where χ_i_ represents the distance of the *i*^th^ atom between the two units, and N represents the number of equivalent atom pairs. In this procedure, only the Cα atom was applied to Eq. (1). The minimum RMSD indicates the global deviation of DPP-4 structure between the two units (the minimum RMSD value will be referred to as RMSD). A graphics software program, PyMOL (Schrödinger, Inc., New York, NY, USA), was used for superposition [28].

[Step 3] Steps 1 and 2 were conducted for every combination. When targeting 147 inhibitor-bound units, there are 10,731 combinations.

[Step 4] After optimum superposition for all combinations was achieved, the average distance of the *i*^th^ Cα atom between two units was calculated using Eq. 2, and defined as the *i*^th^ Cα atom variation between units.

2 $$ {\left[\mathrm{Variation}\kern0.5em \mathrm{between}\kern0.5em \mathrm{units}\right]}_{\kern0.5em i}=\frac{1}{\mathrm{M}}{\displaystyle \sum_k^{\mathrm{M}}{\updelta}_{ik}} $$

where δ_*ik*_ is the distance of the *i*^th^ atom for the *k*^th^ combination between two units, and M represents the number of combinations. When targeting 147 inhibitor-bound units, M is 10,731.

### Side chain variation between units

Side chain variation between units was calculated as follows:

- [Step 1] From the above-mentioned units, two units were selected.
- [Step 2] The two units were superimposed so that the RMSD of main chain (N, Cα and C atoms) of the *i*^th^ residue would be minimized (Eq. 1 was used). The minimum RMSD calculated here represents the main chain deviation of the *i*^th^ residue between the two units.
- [Step 3] Steps 1 and 2 were conducted for every combination.
- [Step 4] After superposition for all combinations, the average distance of a specific side chain atom (e.g., the serine Oγ atom) between two units was calculated using Eq. 2 from the previous procedure. This was defined as the side chain variation of the *i*^th^ residue between units.

### Calculating the exposed surface area

The exposed surface area per one residue of one unit was calculated. In the calculation, water O atoms and the inhibitor were excluded from the unit. The exposed surface area was determined using the Web Server GETAREA (Sealy Center for Structural Biology, University of Texas Medical Branch, Galveston, TX, USA) [29]. For each residue, an average of 147 inhibitor-bound units was calculated (this value is called the exposed surface area).

## Results and discussion

### Cα atom variation

The average RMSD targeting Cα atoms between 147 inhibitor-bound units was 0.48 Å and the maximum RMSD was 0.98 Å. Considering that the minimum resolution of the adopted structures, that is the minimum coordinate error of the structures used, is 1.62 Å (PDB ID: 4A5S, [30]) and that DPP-4 is a large molecule composed of more than 700 residues, we suggest that even the maximum RMSD is below the measurement error range and the units examined are all similar. Keedy et al. reports that the crystal structure of a protein is affected more by cryocooling than by the lab performing the experiment [31]. Eight units (PDB ID: 2AJL_I, 2AJL_J, 2I03_B, 2I78_B, 2OLE_A, 2OLE_B, 3EIO_A and 3EIO_B) were measured in X-ray diffraction at a higher temperature (in the range of 200 K to 298 K) than the other inhibitor-bound units (Additional file 2: Table S1), but the high temperature-measured units were not large in the average RMSD compared with the other units (in the range from 0.46 Å to 0.56 Å). This result suggests that DPP-4 global structure is not changed in the temperature range from 90 to 298 K.

**Fig. 2:**
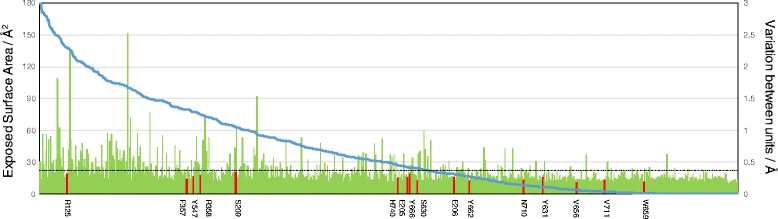
Cα atom variations of DPP-4. Residues in the inhibitor binding area are labeled (red columns). Cα atom variations of residues in the inhibitor binding area are below the average variation of all Cα atoms in DPP-4 (0.37 Å, dashed black line). Cα atoms are listed in the order of their exposed surface area. The vertical axis on the left side is the exposed surface area (blue line graph) and the vertical axis on the right side is the variation value (green bar and red column graph)

The RMSD measures global deviation between units, but cannot identify partial deviation within molecule. Thus, each Cα atom variation between units was calculated and presented in the order of the exposed surface area (Fig. 2). Generally, in a large molecule, the surface (outside) is more variegated than the inside because the former is more affected by the external environment (e.g., a crystal forming condition or measurement temperature) than the latter. The larger the exposed surface area, the more frequently Cα atoms with a large variation were found in the DPP-4 structure. However, all residues in the inhibitor binding area had a less variation than the mean variation of the 724 residues (0.37 Å, Fig. 2), indicating that the main chain structure of the inhibitor binding area was only slightly altered according to the inhibitor used.

### Side chain variation

**Fig. 3:**
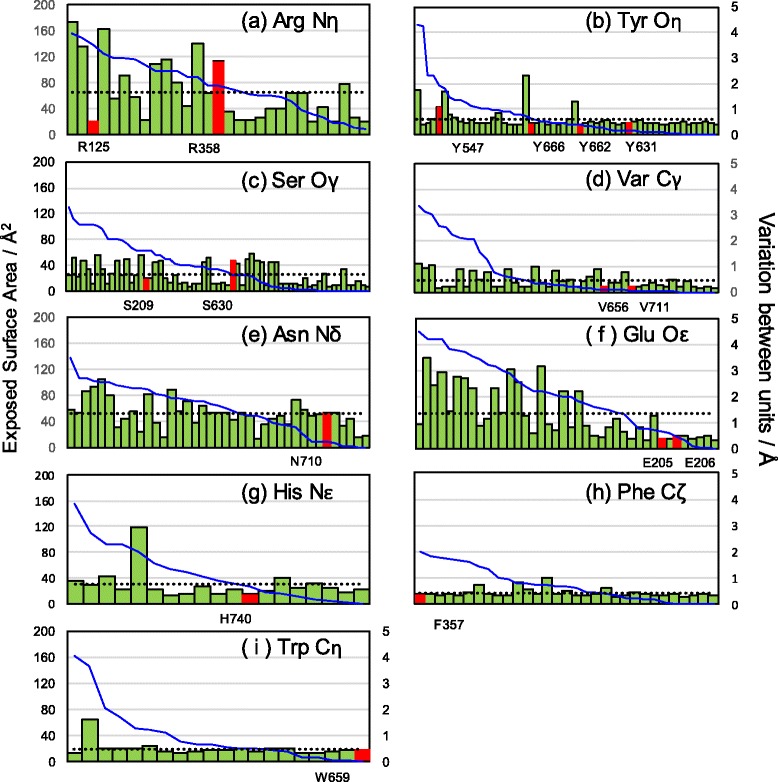
Side chain variations of DPP-4. Sixteen residues in the inhibitor binding area are labeled (red columns). Thirteen residues (Arg125, Tyr 631, Tyr 662, Tyr 666, Ser209, Val656, Val711, Asn710, Glu205, Glu206, His740 and Phe357 and Trp659) have their side chain variations below the average variation of the equivalent amino acids (dashed black line). Specific atoms that are positioned furthest from the main chain were calculated to be the variation. The graph shows the variations for each amino acid and they are listed in the order of the exposed surface area. The vertical axis on the left is the exposed surface area (blue line graph) and the vertical axis on the right is the variation value (green bar and red column graph). The variations of the two Arg Nη atoms, the two Glu Oε atoms and the two Val Cγ atoms are averaged, respectively. **a** Arg Nη atom, **b** Tyr Oη atom, **c** Ser Oγ atom, **d** Val Oγ atom, **e** Asn Nδ atom, **f** Glu Oε atom, **g** His Nε atom, **h** Phe Cζ atom and **i** Trp Cη atom

**Fig. 4:**
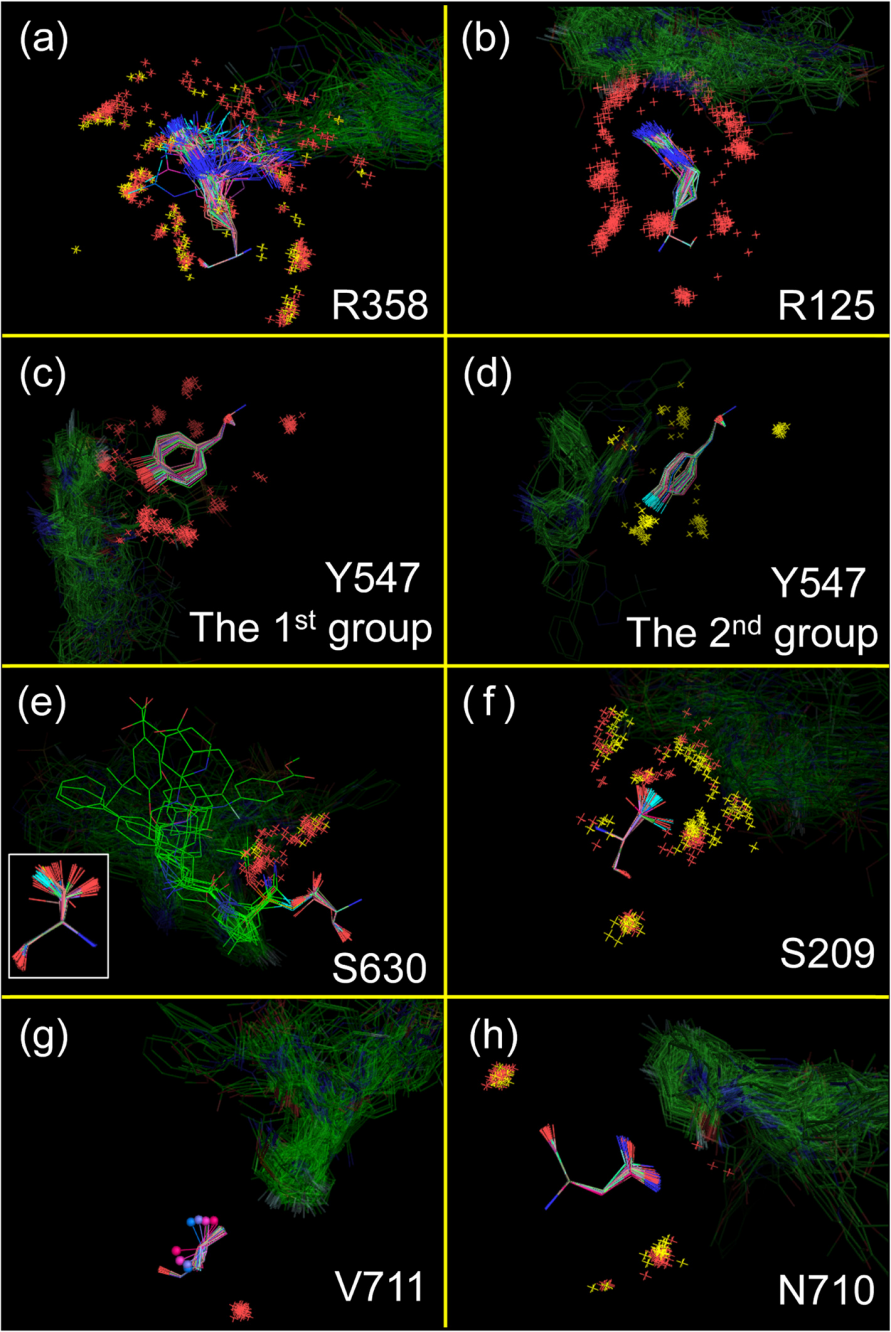
Superposition of some residues in the inhibitor binding area. In each residue, 147 inhibitor-bound units are superimposed so that the RMSD targeting main chain (N, Cα and C atoms) would be minimized based on a specific inhibitor-bound unit. Excluding some exceptions, O and N atoms are colored red and blue, respectively, and the carbon skeleton of inhibitors is shadowed in green. **a** Arg358: The units close to the inhibitor (<4 Å) have the Nη atom colored cyan and the water O atom colored yellow. **b** Arg125. **c** and **d** Tyr547: The Tyr547 side chains were divided into **c** the first and **d** the second group. In the second group, the Oη atom is colored cyan and the water O atom is colored yellow. **e** Ser630: The units in which inhibitor forms a covalent bond have the Oγ atom colored cyan, the inhibitor drawn by a line and the water O atom colored yellow. The image of the different angle is drawn at the lower left in this Figure. **f** Ser209: Some units close to the inhibitor (<4 Å) have the Oγ atom colored cyan and the water O atom colored yellow. **g** Var711: The units in which the Cγ atoms have an opposite direction to the other units have the Cγ atom marked by a “sphere”. **h** Asn710: Some units in which the Oδ and Nδ atoms are switched to each other have the water O atom colored yellow

Side chain variation was classified for each amino acid class and presented in the order of the exposed surface area (Fig. 3). We also visually observed the superposition of 147 inhibitor-bound units (Fig. 4). In the inhibitor binding area, the side chains of Arg358, Tyr547 and Ser630 had a larger variation compared with the average of the equivalent amino acids (Fig. 3a, b and c).

The Arg358 side chains were oriented in a disorderly manner (Fig. 4a). This disorder was found in the inhibitor-free units (Additional file 3: Figure S2a). Arg358 constitutes a part of the S2 extensive subsite, and out of 68, 21 inhibitors (out of 147 inhibitor-bound units, 34 units) were close to this residue (<4 Å). However, there was no trend in the orientation of the Arg358 side chains, even when focusing on only the 34 units (the Nη atoms are colored cyan in Fig. 4a). These results indicate that the Arg358 side chain has no specific orientation regardless of the presence/absence of inhibitor. To inquire into the cause of this large variation, we compared Arg358 with Arg125 whose side chain had the minimum variation (Fig. 4a and b). Arg125 had concentrated distributions of some water O atoms surrounding the whole of residue (Fig. 4b), whereas Arg358 had no concentrated distribution of water O atom surrounding the Nη atoms (Fig. 4a). The exposed surface area of Arg125 is larger than that of Arg358 at first sight, but the Arg125 side chain may be fixed because it is surrounded by some fixed hydrated water molecules. On the other hand, the perimeter of the Arg358 Nη atoms is free from hydrated water, and therefore the orientation of the Arg358 side chain may be flexible.

The Tyr547 benzene rings had their orientation depolarized (Fig. 4c and d). Sheehan et al. reports that the Tyr547 χ^1^ dihedral angle changes by 70° between the two orientations [32]. The Tyr547 benzene rings of the inhibitor-free units showed only one direction (Additional file 3: Figure S2b), and this direction was similar to that of one group of the inhibitor-bound units (Fig. 4c: the first group, the Oη atoms are colored red). The other group (Fig. 4d: the second group, the Oη atoms are colored cyan) had aromatic ring of the inhibitors stacked on the Tyr547 benzene ring. In the commercial drugs, sitagliptin, saxagliptin, trelagliptin, vildagliptin, anagliptin and omarigliptin were the first group, whereas linagliptin and alogliptin were the second group. These results suggest that the Tyr547 benzene ring could shift to a different direction from the original direction observed for the inhibitor-free units to obtain π-π stacking interaction with inhibitor. The π-π stacking interaction of the Tyr547 benzene ring has been reported by many studies [13–16, 30, 32–39]. However, the Tyr547 hydroxyl group in the original direction sometimes electrostatically interacts with the inhibitor’s polar group or with hydrated water [2, 20, 33, 40, 41].

The S630 Oγ atoms were oriented in all directions (Fig. 4e). This disorder was found in the inhibitor-free units (Additional file 3: Figure S2c). Ser630 is the active center of DPP-4 and was positioned within 4 Å from all 68 inhibitors. Some inhibitors have a cyanopyrrolidine, in which the nitrile C atom covalently bonds to the Ser630 Oγ atom. In this case, the Ser630 Oγ atom was naturally oriented toward the inhibitor and very close to the inhibitor nitrile C atom (<3 Å, the Oγ atom is colored cyan in Fig. 4e) (PDB ID: 2AJL [42], 2G5P, 2G5T, 2G63 [33], 2I03 [43], 3BJM (saxagliptin) [20] and 3W2T (vildagliptin) [13]). However, for inhibitors that do not require a covalent bond, the Ser630 Oγ atom seems to be oriented in a disorderly manner. This result suggests that the important thing for DPP-4 inhibitor may be in preventing GLP-1 from approaching Ser630 spatially rather than in deactivating Ser630 directly. Saxagliptin and vildagliptin are commercial covalent bond drugs, but they do not always have a higher potency than the other noncovalent bond commercial drugs (Table 1). This may be because DPP-4 itself slowly hydrolyzes their covalent bond and finally deactivates the covalent bond drugs [44].

The side chains of Ser209, Asn710 and Val711 did not have a significantly large variation compared with the average of the equivalent amino acids (Fig. 3c, d and e), but the superposition of the equivalent amino acids revealed irregular orientational alternatives (Fig. 4f, g and h). In the inhibitor-free units, the Ser209 and Val711 side chains showed the regular orientations (Additional file 3: Figure S2d and e). The Ser209 side chains had no trend when “close to inhibitor” (<4 Å, the Oγ atoms are colored cyan in Fig. 4f) and “not close to inhibitor” (the Oγ atoms are colored red in Fig. 4f) were compared. Val711 had only four units (3O95_B, 3O95_D, 3O9V_B and 3O9V_D) in which the Cγ atoms were irregularly oriented against the other inhibitor-bound units (the Cγ atoms are marked by sphere in Fig. 4g), but this irregularity was found in a PDB (3O95_B and 3O95_D vs. 3O95_A and 3O95_C, 3O9V_B and 3O9V_D vs. 3O9V_A and 3O9V_C) despite having the same inhibitor. The reason that irregular orientations appeared for the Ser209 and Val711 side chains is unclear, although it is possible that the inhibitor influences their orientations.

Asn710 had 47 units in which the Nδ and Oδ atoms were switched to each other compared to the other inhibitor-bound units (Fig. 4h). In the commercial drugs, only linagliptin and alogliptin contained the switched Asn710. This trend was found in the inhibitor-free units (Additional file 3: Figure S2f). There were two fixed water O atoms found in both of the inhibitor-bound/free units, but their positions were not changed by switching the Nδ and Oδ atoms (Fig. 4h). No units had the inhibitor N atom close to (<4 Å) the switched/non-switched Nδ atom of Asn710 (data not shown). This result suggests a tendency that the Nδ and Oδ atoms of Asn710 are switched to each other so that the Nδ atom does not approach too close to the inhibitor’s N atom.

The side chains of the above six residues may be flexible or have orientational alternatives according to the inhibitor used. However, the side chains of the other 10 residues (Arg125, Glu205, Glu206, Phe357, Tyr631, Val656, Trp659, Tyr662, Tyr666 and His740) had a less variation than the average of the equivalent amino acids (Fig. 3), and the superposition of the equivalent amino acids showed that their side chains had little orientational difference between units (data not shown). These results suggest that the side chains of the 10 residues are mostly unaltered according to the inhibitor used. Little movement of the DPP-4 inhibitor binding area through the superposition of some X-ray cocrystal structures with different inhibitors has been described in some studies [35, 41, 45], and they are consistent with our suggestions. The observed rigidity is not a crystal property but probably a DPP-4 property because the collected structures were from different constructs and from different crystal forms, as many researchers have suggested.

### Common binding modes between DPP-4 and inhibitors

We searched electrostatic binding modes between DPP-4 and its inhibitors. In the DPP-4 inhibitor binding area, binding modes of Arg125, Glu205, Glu206, Tyr662 or Asn710 were common between many inhibitors.

**Fig. 5:**
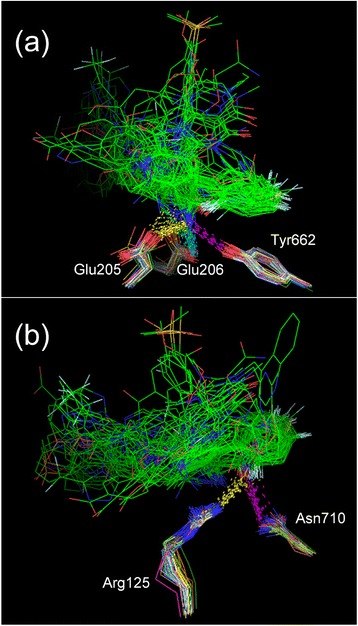
Superposition of DPP4 inhibitors highlights the important binding interactions to some residues. When the inhibitor-bound units were superimposed so that the RMSD targeting Cα atoms in DPP-4 (residue numbers 41–764) would be minimized based on 4PNZ_A, 68 types of inhibitors were simultaneously superimposed. The carbon skeleton of inhibitors is indicated by green line. O, N and halogen atoms are colored red, blue and pale cyan, respectively. **a** Binding modes between Glu205, Glu206 or Tyr662 and inhibitors. The distances between a primary or secondary amino N atom of inhibitor and the Glu205 Oε atom (<4 Å, yellow), the Glu206 Oε atom (<4 Å, cyan), or the Tyr662 Oη atom (<5 Å, magenta) are shown with dashed lines. **b** Binding modes between Arg125 or Asn710 and inhibitors. The distances between an O, N or halogen atom of inhibitor and the Arg125 Nη atom (<4 Å, yellow) or the Asn710 Oδ/Nδ atom (<4 Å, magenta) are shown with dashed lines

Out of 68 inhibitors, 67 had a primary or secondary amino N atom close to one of two Glu205 Oε atoms (<4 Å, dashed yellow lines in Fig. 5a) and one or both of the two Glu206 Oε atoms (<4 Å, dashed cyan lines in Fig. 5a). The exception was 1TKR because the inhibitor in 1TKR has no N atom. In addition, the 67 inhibitors had the same N atom close to the Tyr662 Oη atom (<5 Å, dashed magenta lines in Fig. 5a). This network has been reported to be important for DPP-4 inhibitor to bind to the DPP-4 active center [2, 13–17, 20, 22, 30, 32–37, 40–42, 45–62]. The side chains of the three residues were oriented in a pre-determined direction, as discussed above. This means that a primary or secondary amino group of inhibitor is present without exception at a particular spatial position in DPP-4 and forms binding modes with the Glu205, Glu206 and Tyr662 side chains, suggesting that these binding modes may be a powerful rule for DPP-4 inhibitor to maintain stable binding with DPP-4.

Of 68 inhibitors, 53 had an O, N or halogen atom close to at least one of the Arg125 Nη atoms (Fig. 5b, dashed yellow lines, < 4 Å) and the Asn710 Nδ or Oδ atom (Fig. 5b, dashed magenta lines, < 4 Å). As mentioned above, the Arg125 and Asn710 side chains were oriented in a pre-determined direction, although position switching between the Oδ and Nδ atoms of Asn710 occurred in some units. The network of Asn710 and/or Arg125 also has been described by many authors [20, 32, 34, 35, 40, 41, 49, 52, 55, 56]. However, for the commercial drugs, only linagliptin (PDB ID: 2RGU [16]) had no binding mode with Arg125 and Asn710, and therefore this network may be less important than that with Glu205, Glu206 and Tyr662.

The Ser630 Oγ atom was also close to the polar atom of many inhibitors, but there were minimal common characteristics found on the inhibitor side. The disorderly orientation of the Ser630 Oγ atom was discussed above, suggesting that the placement of an inhibitor with an excluded volume effect close to the Ser630 hydroxyl group is a main role for some DPP-4 inhibitors.

### Common water molecules between units

**Fig. 6:**
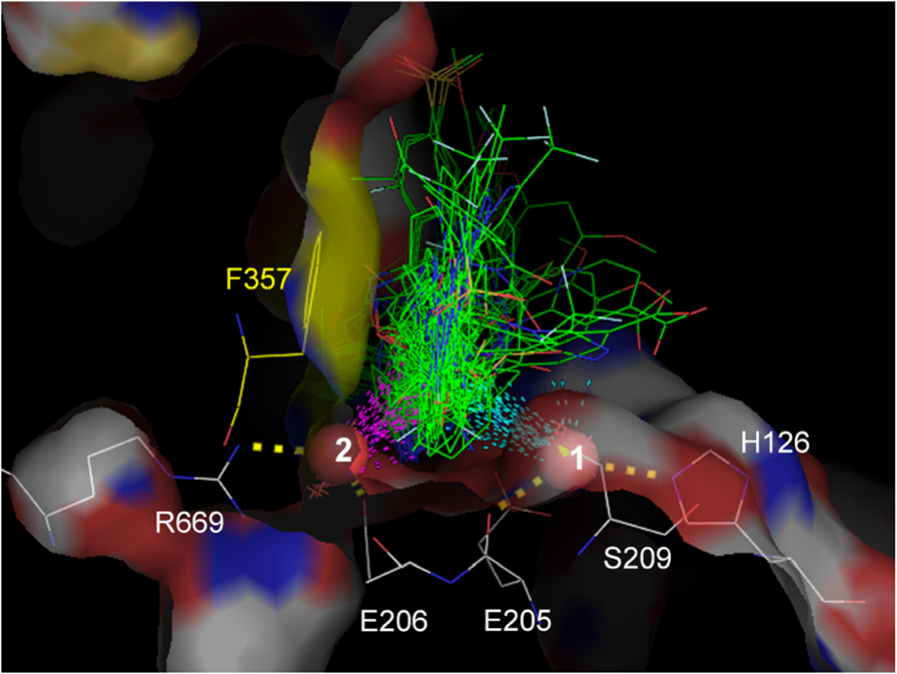
Two specific water O atoms close to inhibitors (red spheres). Both of the two water O atoms were found in 108 inhibitor-bound units. When superimposed so that the RMSD targeting Cα atoms in DPP-4 (residue 41–764) would be minimized based on 1X70_A, water O atoms in the unit were simultaneously superimposed. The carbon skeleton of inhibitors is shown with green line. O, N and halogen atoms are colored red, blue and pale cyan, respectively. The DPP-4 structure was obtained from the coordinates of PDB ID 1X70. The distances between inhibitors and the first specific water O atoms (No. 1) are shown with dashed cyan lines. The distances between inhibitors and the second specific water O atoms (No. 2) are shown with dashed magenta lines. The distances between the two water O atoms and His126, Glu205, Gul206, Ser209 or Arg669 are shown with dashed bold yellow line. Phe357 (yellow area) forms a large wall at the S2 subsite

When two units were superimposed, water O atoms within 1 Å were defined as identical. 2OQI_B, 2OQV_A, 3F8S_A and 3F8S_B had no registered water O atoms. Two hundred thirty-eight water O atoms corresponded to the above identical definition in at least 61 out of the remaining 143 inhibitor-bound units. Among these, 26 water O atoms were close to the inhibitor binding area (<4 Å), and two specific water O atoms were also close to many inhibitors (<5 Å, red spheres in Fig. 6).

**Fig. 7:**
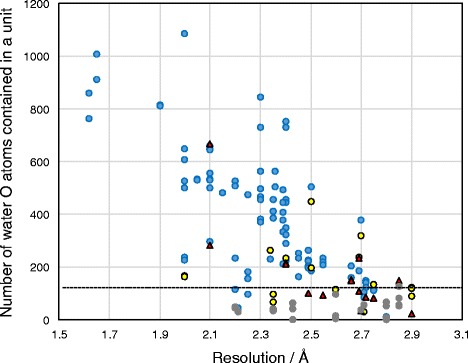
Correlation between X-ray diffraction resolution and the number of water O atoms registered. Ninety-two units (blue circles) have both of the two specific water O atoms. Sixteen units (red triangles) have only the first specific water O atom (the first red sphere in Fig. 6), and 16 units (yellow circles) have only the second specific water O atom (the second red sphere in Fig. 6). Nineteen units (grey circles) have neither of the two water O atoms, and 2OQI_B, 2OQV_A, 3F8S_A and 3F8S_B (grey circles) have no registered water O atoms. The unit that contains the most water O atoms in the above 19 units (grey circles) is PDB ID 3O95_B (129 water O atoms, dashed line)

The first specific water O atom was observed in 108 inhibitor-bound units (blue circles and red triangles in Fig. 7). It was close to 52 inhibitors (<5 Å, dashed cyan lines in Fig. 6), the His126 Nε, the Glu205 Oε and the Ser209 Oγ atoms (<4 Å, dashed bold yellow lines in Fig. 6). This water molecule has been described in some studies [13, 45, 63]. The second specific water O atom was also observed in 108 units (blue and yellow circles in Fig. 7). It was close to 55 inhibitors (<5 Å, dashed magenta lines in Fig. 6), the Glu206 Oε and the Arg669 Nη atoms (<4 Å, dashed bold yellow lines in Fig. 6). Ninety-two units had both of the two water O atoms (blue circles in Fig. 7).

Many of the inhibitor-bound units that lack either of the two specific water O atoms were distributed over 2.5 Å resolution (red triangles, and yellow and grey circles in Fig. 7). Nineteen units that did not have the two O water atoms (grey circles in Fig. 7) showed few registered water O atoms (the maximum was 129 water O atoms, dashed line in Fig. 7). These results suggest that the two specific water O atoms were overlooked in the units because their positions could not be identified accurately. Keedy el al. reports that the ordering of surface-associated waters is driven by cryocooling [31]. However, the high temperature-measured units had two water O atoms in the same position as the other inhibitor-bound units (Additional file 4: Figure S3), and the two specific water O atoms were also found in the inhibitor-free units (Additional file 5: Figure S4). The B-factor is an index of atom thermal mobility within crystal structure. In many inhibitor-bound units, both of the two specific water O atoms had a lower B-factor than the average B-factors of all water O atoms and of all protein heavy atoms (Additional file 6: Table S2 and Additional file 7: Table S3). Generally, hydrated water that is usually entrapped from local protein structure has a lower B-factor than crystal water that is accidentally entrapped by crystallization. The above two water molecules we found seem to exist as hydrated water that is taken into the local position in inherent DPP-4, independently of a temperature change (ranged from 90 to 298 K) and regardless if DPP-4 has an inhibitor.

The primary role of the two specific water molecules (probably hydrated water) may be forming an electrostatic network with His126, Glu205 and Ser209, and with Glu206 and Arg669, and maintaining the orientation of the side chains of Glu205 and Glu206 through the network may be especially important for inhibitor binding. The adamantan hydroxyl group of vildagliptin is reported to interact with the first specific water molecule [13], and it may be important for the potency of vildagliptin. The two water molecules, however, do not seem to have an electrostatic interaction with many inhibitors because they are close to the carbon skeleton of the inhibitors rather than to their polar atoms. All 68 inhibitors were placed as if they were avoiding the position of the two water O atoms. The secondary role of the two water molecules may be an excluded volume effect, such as arranging inhibitor appropriately at the S2 subsite. The two water O atoms were located at the bottom of the S2 subsite, where Phe357 formed a large wall at the S2 subsite (yellow area in Fig. 6). The hydrophobic or π-π stacking interaction of the Phe357 benzene ring has been reported to be important for binding in many inhibitors [2, 17, 22, 33, 42, 45, 47–49, 51–55, 57, 60, 64]. Further research is needed to determine the detailed relationship between the two specific water molecules and inhibitor potency. However, the prearrangement of the two water molecules should be considered in *in silico* screening not to lead inhibitor to improper position.

It has been often asked whether hydrated water in a crystal structure is applicable in solution. Generally, crystal and hydrated water occupies an average of 50 % of the crystal structure, and many protein crystal structures have been reported to reflect the structure in a water solution [65–77]. A molecular dynamics simulation showed that the frequent appearance positions of water molecule during the simulation agrees with the hydrated water site in the X-ray crystal structure [78]. However, whether the two specific water molecules are necessary for an inhibitor to maintain stable binding with DPP-4 should be confirmed by a solution structure using nuclear magnetic resonance (NMR) or molecular dynamics simulation (the solution structure of DPP-4 has not yet been obtained using NMR). A few molecular dynamics simulations have reported the interaction between DPP-4 and its inhibitor [79, 80], but further simulations evaluating hydrated water require additional research, which we plan to perform in the future.

### DPP-4 inhibitory affinity of the inhibitor used

The results so far were provided based on only crystal structures whose resolutions were less than 3.0 Å to provide high-quality information (Additional file 2: Table S1). However, if the results are used by *in silico* screening, a realistic potency should accompany the candidates that passed the screening test. DPP-4 inhibitory activity of PDBs (inhibitors) that we collected ranged from 0.1 to 30,000 nM in IC_50_/Ki, and many of them had high inhibitory activity of less than 30 nM (Additional file 8: Table S4) [24, 25]. This value is a standard of the commercial drugs, as shown by Table 1. Thus, we used high-quality resources in regard to resolution and DPP-4 inhibitory affinity. Therefore, our study provides necessary information for *in silico* screening, and may provide a necessary minimum consensus to help in the discovery of a novel DPP-4 inhibitor that is commercially useful. However, DPP-4 inhibitor also needs to meet some optional requirements to acquire substantial commercial-drug level inhibitory activity, e.g. geometric fitness and electrostatic/non-electrostatic interaction with some subsites.

## Conclusions

To clarify whether DPP-4 alters its general or partial structure according to the inhibitor used and whether DPP-4 has a common rule for inhibitor binding, we comprehensively analyzed X-ray cocrystal structures of DPP-4 and its inhibitor. Before beginning this research, we expected that DPP-4 would alter at least its partial structure because DPP-4 can bind to inhibitors of various shapes. However, our results were different from our initial expectations. All the main and side chains in the inhibitor binding area were only minimally altered except for some side chains (Ser209, Arg358, Tyr547, Ser630, Val656 and Asn710), despite binding to inhibitors of various shapes. Some residues (Arg125, Glu205, Glu206, Tyr662 and Asn710) in the area had binding modes to fix a specific atom of inhibitor to a particular spatial position in DPP-4. We found two specific water molecules that are common to 92 DPP-4 crystal structures. The two water molecules were close to many inhibitors, and seemed to play two roles: maintaining the orientation of the Glu205 and Glu206 side chains, which is important for inhibitor binding through an electrostatic network via the two water molecules, and arranging the inhibitor appropriately at the S2 subsite. In this study, we used high-quality resources, and therefore this information may provide a necessary minimum consensus to help in the discovery of a novel DPP-4 inhibitor that is commercially useful.

## Abbreviations

DPP-4, dipeptidyl peptidase IV; GLP-1, glucagon-like peptide-1; IC_50_, half maximal inhibitory concentration; Ki, inhibition constant; nM, nanomolar; NMR, nuclear magnetic resonance; PDB, Protein Data Bank; RMSD, root mean square deviation

## Additional files

Additional file 1:
**Figure S1.** Structural formula and PDB ID of 68 types of DPP-4 inhibitors. The PDB ID is followed by the chain ID that we used in this study. (DOCX 711 kb) Additional file 2:
**Table S1.** Resolution and measured temperature of X-ray crystal structural analysis. (DOCX 29 kb) Additional file 3:
**Figure S2.** Superposition of some residues in ligand-free units. Eight ligand-free units are superimposed so that the RMSD targeting the main chain (the N, Cα and C atoms) would be minimized based on a specific ligand-free unit. **a** Arg358, **b** Tyr547, **c** Ser630, **d** Ser209, **e** Val711 and **f** Asn710. (DOCX 124 kb) Additional file 4:
**Figure S3.** Superposition of two specific water O atoms in high temperature-measured units (yellow symbol “+”). The temperature-measured units were superimposed so that Cα atoms in DPP-4 (residue numbers 41–764) would be minimized based on 1X70_A (the two specific water O atoms of 1X70_A are marked by red symbol “+” in the white broken circles). The two specific water O atoms registered in the high temperature-measured units are superimposed well onto those in 1X70_A that was measured at low temperature (yellow symbol “+” in the white broken circles). (DOCX 273 kb) Additional file 5:
**Figure S4.** Superposition of two specific water O atoms in ligand-free units (yellow spheres). Six ligand-free units (1J2E_A, 1J2E_B, 1NU6_A, 1PFQ_A, 1TK3_A and 1TK3_B) that had one of the two specific water O atoms were superimposed so that Cα atoms in DPP-4 (residue numbers 41–764) would be minimized based on 1X70_A. The two specific water O atoms of the six ligand-free units (yellow spheres in the white broken circles) superimposed well onto those in 1X70_A with ligand (red spheres). (DOCX 329 kb) Additional file 6:
**Table S2.** B-factor of the first specific water O atom in a unit. (DOCX 40 kb) Additional file 7:
**Table S3.** B-factor of the second specific water O atom in a unit. (DOCX 41 kb) Additional file 8:
**Table S4.** DPP-4 inhibitory activity. (DOCX 30 kb)
