# Supplementary material for: Comprehensive analysis of the Co-structures of dipeptidyl peptidase IV and its inhibitor
Source: BMC Struct Biol. 2016 Aug 5;16:11. doi: 10.1186/s12900-016-0062-8 (PMC4974693; doi:10.1186/s12900-016-0062-8)
Supplement: Additional file 1: — Figure S1. Structural formula and PDB ID of 68 types of DPP-4 inhibitors. The PDB ID is followed by the chain ID that we used in this study. (DOCX 711 kb) [file 12900_2016_62_MOESM1_ESM.docx]

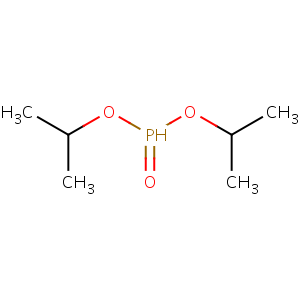

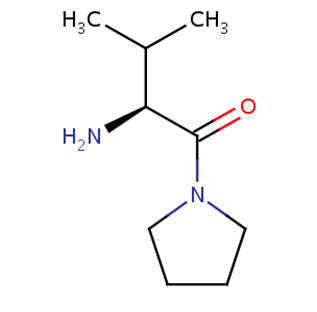

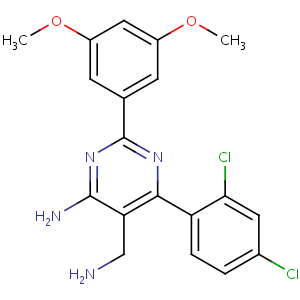


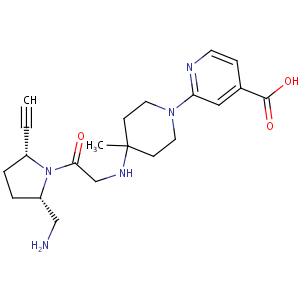

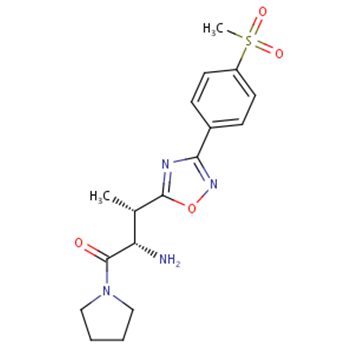

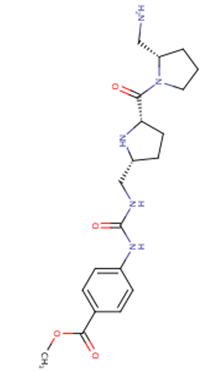
[
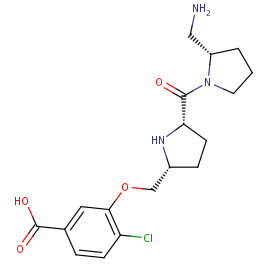
](http://www.rcsb.org/pdb/images/ACF_600.gif)
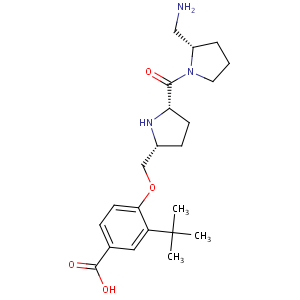

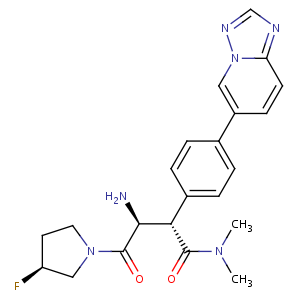

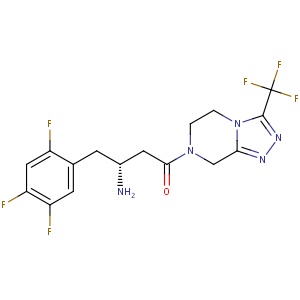

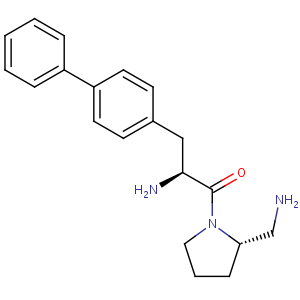

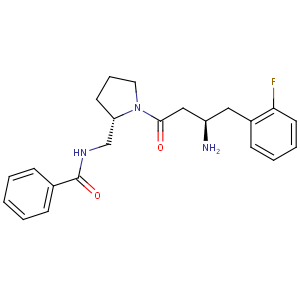


2BUB A and B chains

2AJL I and J chains

2G63 B chain

2HHA A and B chains

**Sitagliptin**

1X70 A and B chains

**Figure S1.**

2I03 B chain

2FJP A and B chains

2G5P A chain

1TKR A and B chains

1RWQ A and B chains

1N1M A and B chains Achina

2G5T A chain


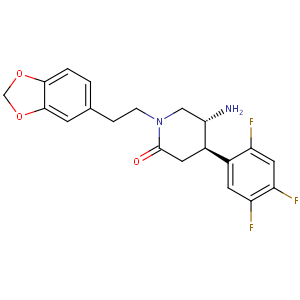

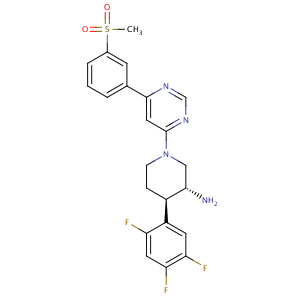

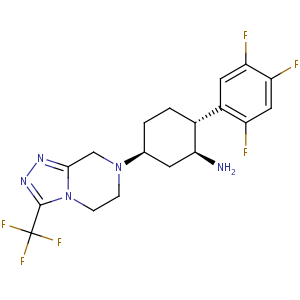

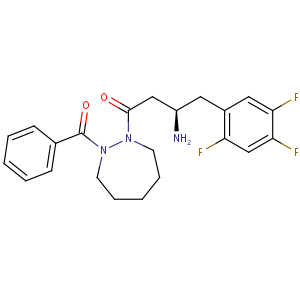

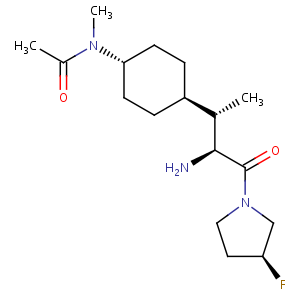

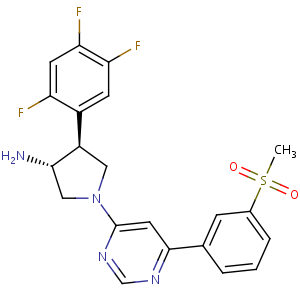

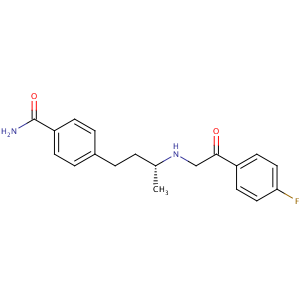

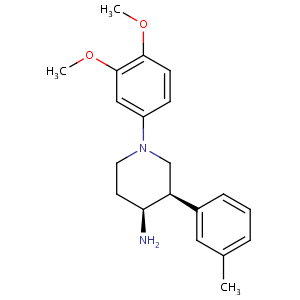

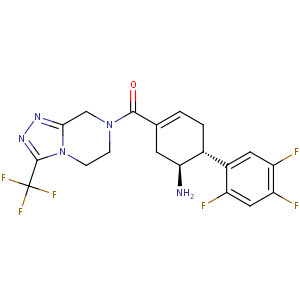

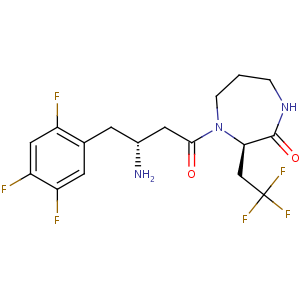

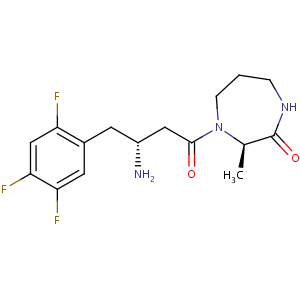

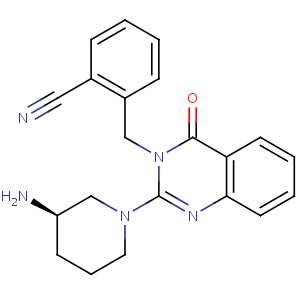


2JID A and B chains

2OGZ A and B chains

2OAG B chain

2OQI B chain

2OQV A chain

2P8S A and B chains

2OPH A and B chains

2ONC A, B, C and D chains

2OLE A and B chains

2IIV A and B chains

2I78 B chain

2IIT A and B chains


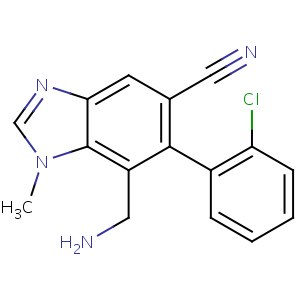

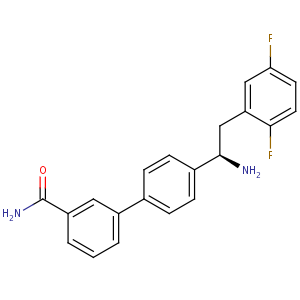

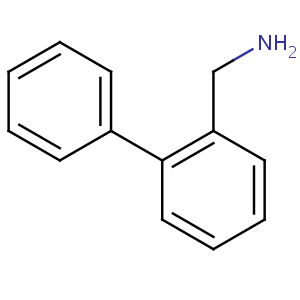

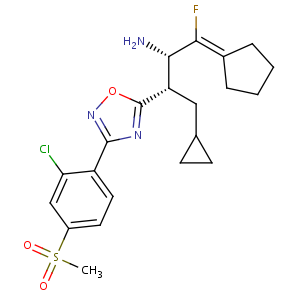

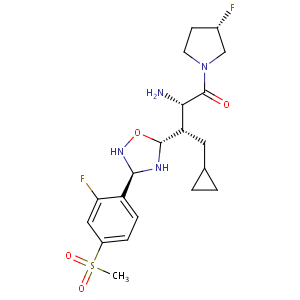

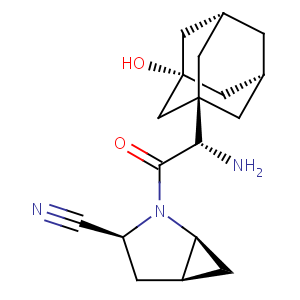

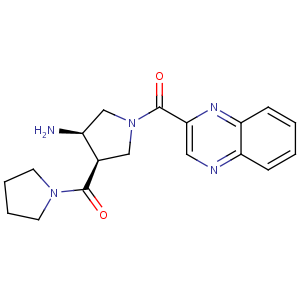

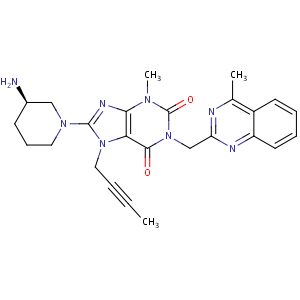

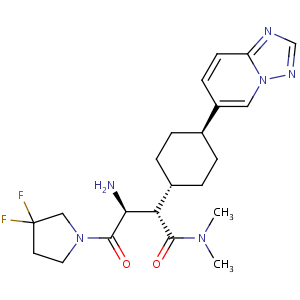

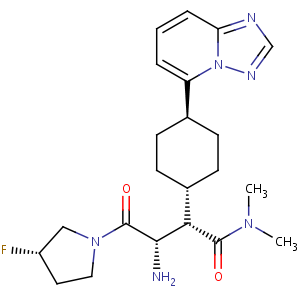

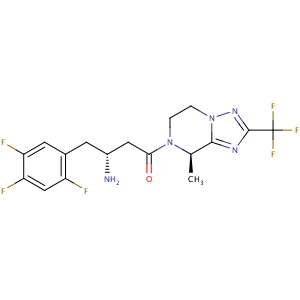

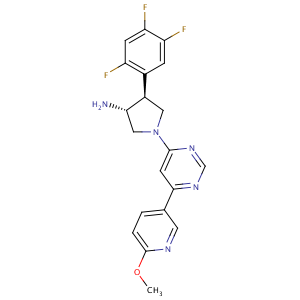


3C43 A and B chains

**Linagliptin**

2RGU A and B chains

2RIP A chain

2QTB A and B chains

**Saxagliptin**

3BJM A and B chains

3C45 A and B chains

3CCB A, B, C

and D chains

3D4L A and B chains

3CCC A, B, C

and D chains

2QT9 A and B chains

2QOE A and B chains

2QJR A and B chains


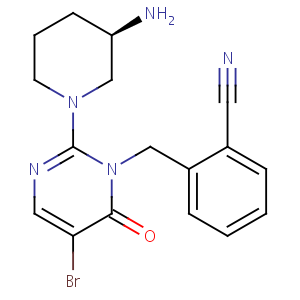

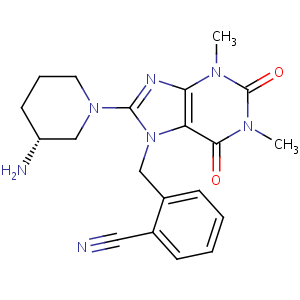

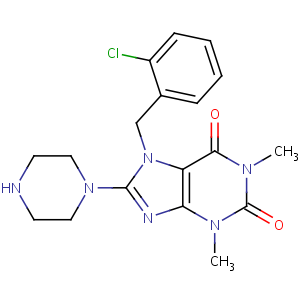

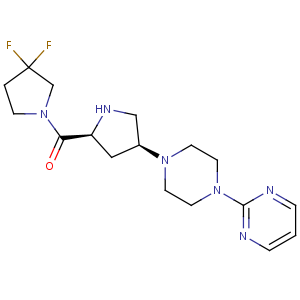

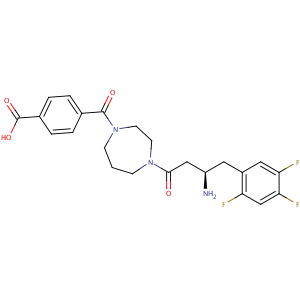

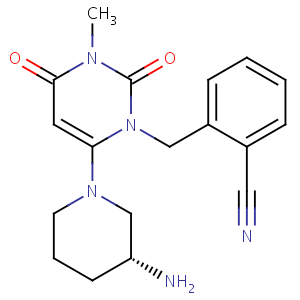


**Alogliptin**

3G0B A, B, C

and D chains

3F8S A and B chains

3EIO A and B chains


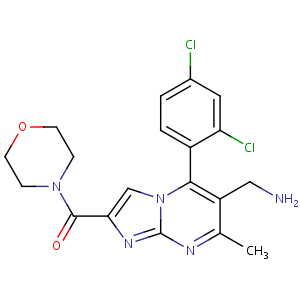

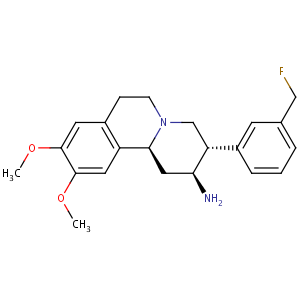

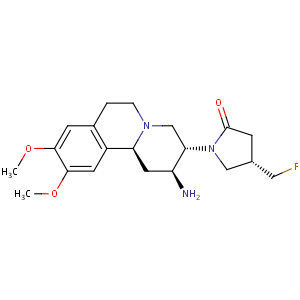

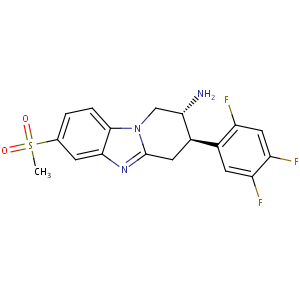

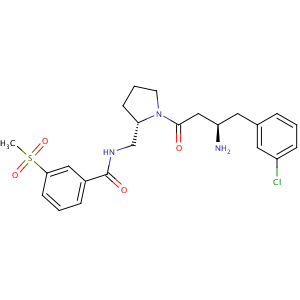

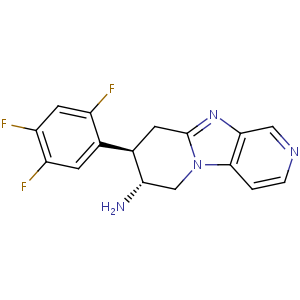

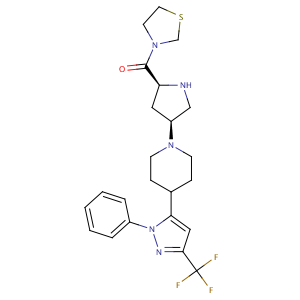

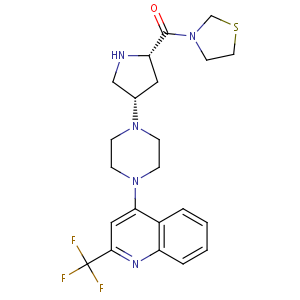

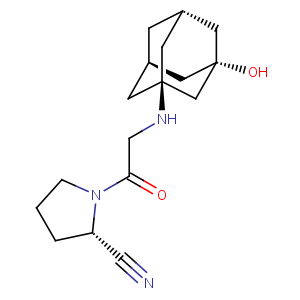

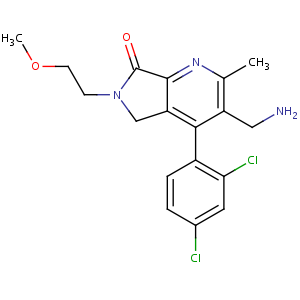

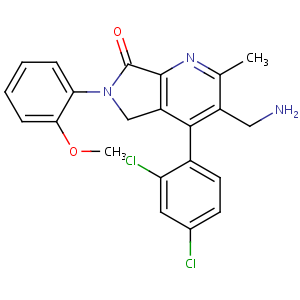

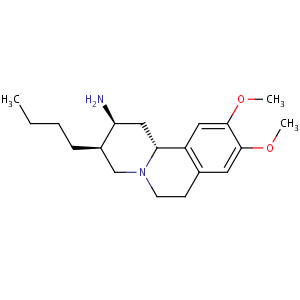

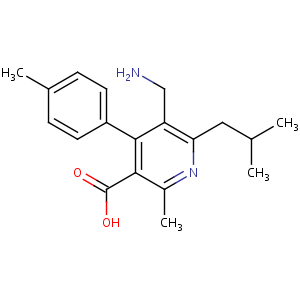

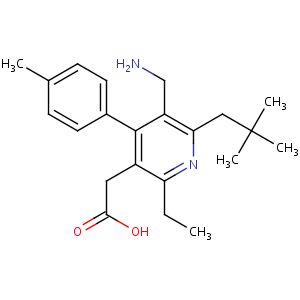

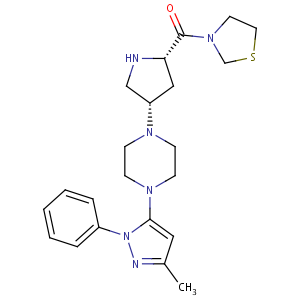

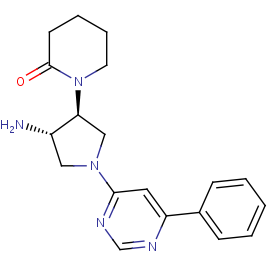

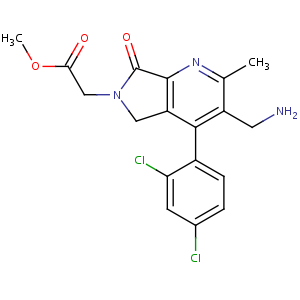

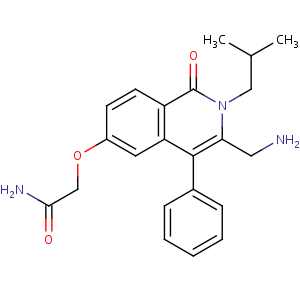


3HAC A and B chains

3H0C A, and B chains

3KWF A and B chains

3KWJ A and B chains

3NOX A and B chains

3HAB A and B chains

3G0G A, B

and C chains

3G0D A, B, C

and D chains

3G0C A, B, C

and D chains

3VJL A and B chains

3SX4 A and B chains

3OPM A, B, C and D chains

**Vildagliptin**

3W2T A and B chains

3VJM A and B chains

**Teneligliptin**

3VJK A and B chains

3SWW A and B chains

3Q0T A and B chains

3QBJ A and B chains

3OC0 A and B chains

3O9V A, B, C and D chains

3O95 A, B, C and D chains

[
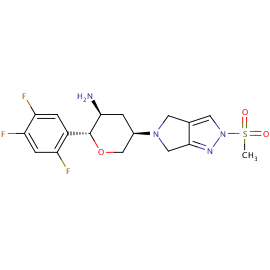
](http://www.rcsb.org/pdb/images/2VH_600.gif)[
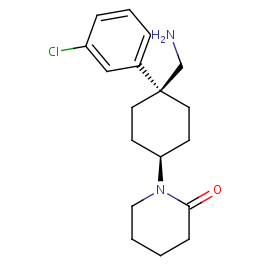
](http://www.rcsb.org/pdb/images/2KV_600.gif)[
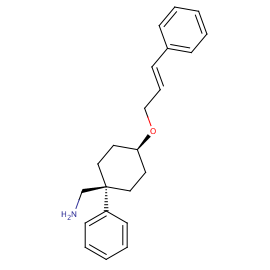
](http://www.rcsb.org/pdb/images/2KS_600.gif)
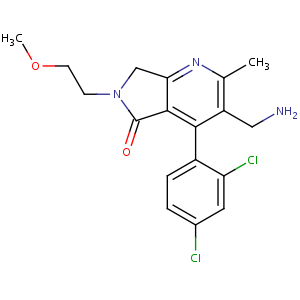

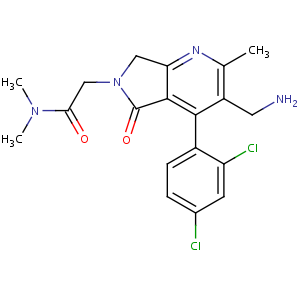
[
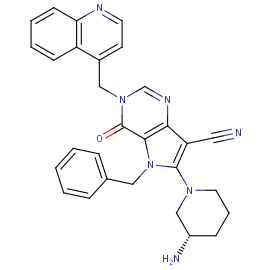
](http://www.rcsb.org/pdb/images/N7F_600.gif)
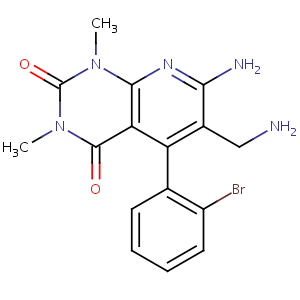
[
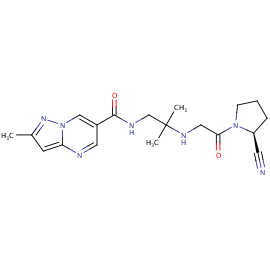
](http://www.rcsb.org/pdb/images/SKK_600.gif)

**Omarigliptin**

4PNZ A and B chains

4N8D A and B chains

4G1F A, B and C chains

4JH0 A and B chains

4A5S A and B chains

**Anagliptin**

3WQH A and B chains

4LKO A and B chains

4N8E A and B chains
