# Supplementary material for: Comprehensive analysis of the Co-structures of dipeptidyl peptidase IV and its inhibitor
Source: BMC Struct Biol. 2016 Aug 5;16:11. doi: 10.1186/s12900-016-0062-8 (PMC4974693; doi:10.1186/s12900-016-0062-8)
Supplement: Additional file 2: — Table S1. Resolution and measured temperature of X-ray crystal structural analysis. (DOCX 29 kb) [file 12900_2016_62_MOESM2_ESM.docx]

**Table S1.** Resolution and measured temperature of X-ray crystal structural analysis.

| PDB ID | Resolution / Å | Measured temperature / K |
| --- | --- | --- |
| (ligand-bond) |  |  |
| 1N1M | 2.50 | 100 |
| 1RWQ | 2.20 | 90 |
| 1TKR | 2.70 | 100 |
| 1X70 (Sitagliptin) | 2.10 | 100 |
| 2AJL | 2.50 | 200 |
| 2BUB | 2.66 | 100 |
| 2FJP | 2.40 | 100 |
| 2G5P | 2.40 | 100 |
| 2G5T | 2.30 | unknown |
| 2G63 | 2.00 | 100 |
| 2HHA | 2.35 | 100 |
| 2I03 | 2.40 | 200 |
| 2I78 | 2.50 | 200 |
| 2IIT | 2.35 | 100 |
| 2IIV | 2.15 | 100 |
| 2JID | 2.80 | 100 |
| 2OAG | 2.30 | unknown |
| 2OGZ | 2.10 | 100 |
| 2OLE | 2.40 | 295 |
| 2ONC | 2.55 | 100 |
| 2OPH | 2.40 | 100 |
| 2OQI | 2.80 | 100 |
| 2OQV | 2.80 | 100 |
| 2P8S | 2.20 | 100 |
| 2QJR | 2.20 | 100 |
| 2QOE | 2.30 | 100 |
| 2QT9 | 2.10 | 100 |
| 2QTB | 2.25 | 100 |
| 2RGU (Linagliptin) | 2.60 | 100 |
| 2RIP | 2.90 | 100 |
| 3BJM (Saxagliptin) | 2.35 | 100 |
| 3C43 | 2.30 | 100 |
| 3C45 | 2.05 | 100 |
| 3CCB | 2.49 | unknown |
| 3CCC | 2.71 | unknown |
| 3D4L | 2.00 | 100 |
| 3EIO | 2.00 | 298 |
| 3F8S | 2.43 | 100 |
| 3G0B (Alogliptin) | 2.25 | 100 |
| 3G0C | 2.69 | 100 |
| 3G0D | 2.39 | 100 |
| 3G0G | 2.45 | 100 |
| 3H0C | 2.66 | unknown |
| 3HAB | 2.10 | 100 |
| 3HAC | 2.00 | 100 |
| 3KWF | 2.40 | unknown |
| 3KWJ | 2.80 | unknown |
| 3NOX | 2.34 | 100 |
| 3O95 | 2.85 | 92 |
| 3O9V | 2.75 | 92 |
| 3OC0 | 2.70 | 90 |
| 3OPM | 2.72 | 92 |
| 3Q0T | 2.40 | unknown |
| 3QBJ | 2.21 | 100 |
| 3SWW | 2.00 | 100 |
| 3SX4 | 2.60 | 100 |
| 3VJK (Teneligliptin) | 2.49 | 100 |
| 3VJL | 2.39 | 100 |
| 3VJM | 2.10 | 100 |
| 3W2T (Vildagliptin) | 2.36 | 100 |
| 3WQH (Anagliptin) | 2.85 | 100 |
| 4A5S | 1.62 | 100 |
| 4G1F | 2.90 | 100 |
| 4JH0 | 2.35 | 100 |
| 4LKO | 2.43 | 100 |
| 4N8D | 1.65 | 100 |
| 4N8E | 2.30 | 100 |
| 4PNZ (Omarigliptin) | 1.90 | 100 |
| (ligand-free) |  |  |
| 1J2E | 2.60 | 100 |
| 1NU6 | 2.10 | 90 |
| 1PFQ | 1.90 | unknown |
| 1TK3 | 2.00 | 100 |
