# Supplementary material for: Comprehensive analysis of the Co-structures of dipeptidyl peptidase IV and its inhibitor
Source: BMC Struct Biol. 2016 Aug 5;16:11. doi: 10.1186/s12900-016-0062-8 (PMC4974693; doi:10.1186/s12900-016-0062-8)
Supplement: Additional file 4: — Figure S3. Superposition of two specific water O atoms in high temperature-measured units (yellow symbol “+”). The temperature-measured units were superimposed so that Cα atoms in DPP-4 (residue numbers 41–764) would be minimized based on 1X70_A (the two specific water O atoms of 1X70_A are marked by red symbol “+” in the white broken circles). The two specific water O atoms registered in the high temperature-measured units are superimposed well onto those in 1X70_A that was measured at low temperature (yellow symbol “+” in the white broken circles). (DOCX 273 kb) [file 12900_2016_62_MOESM4_ESM.docx]

**Figure S3.**
