# Supplementary material for: Comprehensive analysis of the Co-structures of dipeptidyl peptidase IV and its inhibitor
Source: BMC Struct Biol. 2016 Aug 5;16:11. doi: 10.1186/s12900-016-0062-8 (PMC4974693; doi:10.1186/s12900-016-0062-8)
Supplement: Additional file 6: — Table S2. B-factor of the first specific water O atom in a unit. (DOCX 40 kb) [file 12900_2016_62_MOESM6_ESM.docx]

**Table S2.** B-factor of the first specific water O atom in a unit.

| unit | B-factor of the first specific water O atom (1) | Average B-factor of all water O atoms in a unit (2) | Difference  [(1)-(2)]* | Average B-factor of all heavy atoms of DPP-4 in a unit (3) | Difference  [(1)-(3)]* |
| --- | --- | --- | --- | --- | --- |
| 1N1M_A | 15.68 | 30.47 | -14.79 | 31.43 | -15.75 |
| 1N1M_B | 29.59 | 30.79 | -1.20 | 34.15 | -4.56 |
| 1TKR_A | 18.21 | 22.49 | -4.28 | 24.44 | -6.23 |
| 1TKR_B | 24.07 | 23.49 | **0.58** | 27.47 | -3.4 |
| 1X70_A (Sitagliptin) | 22.69 | 33.13 | -10.44 | 26.34 | -3.65 |
| 1X70_B (Sitagliptin) | 20.89 | 33.92 | -13.03 | 27.13 | -6.24 |
| 2AJL_I | 18.12 | 14.52 | **3.60** | 22.01 | -3.89 |
| 2AJL_J | 2.61 | 14.97 | -12.36 | 20.55 | -17.94 |
| 2BUB_A | 42.6 | 29.06 | **13.54** | 38.71 | **3.89** |
| 2FJP_A | 26.94 | 32.38 | -5.44 | 33.03 | -6.09 |
| 2FJP_B | 21.75 | 32.70 | -10.95 | 33.42 | -11.67 |
| 2G5P_A | 26.19 | 42.94 | -16.75 | 41 | -14.81 |
| 2G5T_A | 9.37 | 37.12 | -27.75 | 30.32 | -20.95 |
| 2G63_B | 17.81 | 43.66 | -25.85 | 32.65 | -14.84 |
| 2HHA_A | 10.39 | 28.28 | -17.89 | 25.5 | -15.11 |
| 2HHA_B | 17.57 | 29.15 | -11.58 | 26.15 | -8.58 |
| 2I03_B | 25.79 | 51.70 | -25.91 | 47.83 | -22.04 |
| 2I78_B | 21.65 | 26.76 | -5.11 | 30.87 | -9.22 |
| 2IIT_A | 28.66 | 31.54 | -2.88 | 29.55 | -0.89 |
| 2IIT_B | 22.9 | 32.02 | -9.12 | 30.31 | -7.41 |
| 2IIV_A | 16.88 | 29.28 | -12.40 | 26.26 | -9.38 |
| 2IIV_B | 19.62 | 30.28 | -10.66 | 26.96 | -7.34 |
| 2OAG_B | 20.88 | 41.26 | -20.38 | 37.51 | -16.63 |
| 2OGZ_B | 27.23 | 37.02 | -9.79 | 34.71 | -7.48 |
| 2OLE_A | 23.42 | 30.54 | -7.12 | 31.32 | -7.9 |
| 2OLE_B | 11.68 | 28.04 | -16.36 | 30.35 | -18.67 |
| 2ONC_A | 24.11 | 42.70 | -18.59 | 48.85 | -24.74 |
| 2ONC_B | 41.06 | 45.03 | -3.97 | 51.49 | -10.43 |
| 2ONC_C | 31.85 | 45.00 | -13.15 | 51.5 | -19.65 |
| 2OPH_A | 20.28 | 27.92 | -7.64 | 25.49 | -5.21 |
| 2OPH_B | 30.76 | 28.82 | **1.94** | 25.93 | **4.83** |
| 2P8S_A | 20.86 | 31.89 | -11.03 | 27.18 | -6.32 |
| 2P8S_B | 19.76 | 32.34 | -12.58 | 28.25 | -8.49 |
| 2QJR_A | 52.45 | 54.11 | -1.66 | 56.73 | -4.28 |
| 2QJR_B | 40.34 | 52.24 | -11.90 | 56.7 | -16.36 |
| 2QOE_A | 17.74 | 27.61 | -9.87 | 28.39 | -10.65 |
| 2QOE_B | 16.27 | 28.02 | -11.75 | 28.51 | -12.24 |
| 2QT9_A | 19.33 | 29.00 | -9.67 | 23.17 | -3.84 |
| 2QTB_A | 21.2 | 28.84 | -7.64 | 26.23 | -5.03 |
| 2QTB_B | 21.05 | 29.52 | -8.47 | 26.25 | -5.2 |
| 2RGU_B (Linagliptin) | 44.65 | 44.18 | **0.47** | 50.18 | -5.53 |
| 3BJM_A (Saxagliptin) | 33.61 | 40.55 | -6.94 | 41.15 | -7.54 |
| 3BJM_B (Saxagliptin) | 30 | 39.66 | -9.66 | 38.95 | -8.95 |
| 3C43_A | 16.01 | 29.12 | -13.11 | 27.69 | -11.68 |
| 3C43_B | 17.2 | 29.98 | -12.78 | 28.14 | -10.94 |
| 3C45_A | 19.36 | 32.35 | -12.99 | 27.52 | -8.16 |
| 3C45_B | 20.14 | 33.00 | -12.86 | 28.13 | -7.99 |
| 3CCB_A | 24.62 | 39.72 | -15.10 | 50.4 | -25.78 |
| 3CCB_B | 38.78 | 41.60 | -2.82 | 49.38 | -10.6 |
| 3CCB_C | 36.57 | 41.49 | -4.92 | 50.98 | -14.41 |
| 3CCC_A | 40.37 | 43.28 | -2.91 | 53.52 | -13.15 |
| 3CCC_B | 37.5 | 40.73 | -3.23 | 51.6 | -14.1 |
| 3CCC_D | 62.08 | 44.92 | **17.16** | 69.32 | -7.24 |
| 3D4L_A | 25 | 36.39 | -11.39 | 31.39 | -6.39 |
| 3D4L_B | 25.65 | 37.32 | -11.67 | 32.32 | -6.67 |
| 3EIO_A | 18.49 | 23.52 | -5.03 | 33.25 | -14.76 |
| 3EIO_B | 17.55 | 24.00 | -6.45 | 30.53 | -12.98 |
| 3G0B_A (Alogliptin) | 27.38 | 39.53 | -12.15 | 51.76 | -24.38 |
| 3G0B_B (Alogliptin) | 37.37 | 41.91 | -4.54 | 51.34 | -13.97 |
| 3G0B_C (Alogliptin) | 37.76 | 43.32 | -5.56 | 53.58 | -15.82 |
| 3G0B_D (Alogliptin) | 29.99 | 40.73 | -10.74 | 52.24 | -22.25 |
| 3G0C_A | 30.5 | 40.81 | -10.31 | 47.8 | -17.3 |
| 3G0C_B | 22.86 | 42.11 | -19.25 | 47.36 | -24.5 |
| 3G0C_C | 35.28 | 42.28 | -7.00 | 48.21 | -12.93 |
| 3G0D_A | 28 | 47.49 | -19.49 | 51.69 | -23.69 |
| 3G0D_B | 37.34 | 49.11 | -11.77 | 51.74 | -14.4 |
| 3G0D_C | 31.43 | 48.65 | -17.22 | 52.24 | -20.81 |
| 3G0D_D | 43.62 | 51.88 | -8.26 | 56.83 | -13.21 |
| 3G0G_A | 40.74 | 41.55 | -0.81 | 46.44 | -5.7 |
| 3G0G_B | 34.93 | 43.86 | -8.93 | 47.61 | -12.68 |
| 3G0G_C | 24.4 | 43.70 | -19.30 | 51.08 | -26.68 |
| 3H0C_A | 41.26 | 33.03 | **8.23** | 35.92 | **5.34** |
| 3HAB_A | 15.33 | 29.09 | -13.76 | 25.88 | -10.55 |
| 3HAB_B | 17.31 | 29.94 | -12.63 | 25.97 | -8.66 |
| 3HAC_A | 22.89 | 36.27 | -13.38 | 34.66 | -11.77 |
| 3HAC_B | 26.68 | 36.31 | -9.63 | 34.68 | -8 |
| 3KWF_B | 29.74 | 36.43 | -6.69 | 40.1 | -10.36 |
| 3KWJ_B | 31.85 | 22.77 | **9.08** | 46.11 | -14.26 |
| 3NOX_B | 25.07 | 41.81 | -16.74 | 40.4 | -15.33 |
| 3O9V_A | 25.89 | 35.99 | -10.10 | 48.69 | -22.8 |
| 3O9V_B | 34.59 | 34.93 | -0.34 | 48.16 | -13.57 |
| 3O9V_D | 30.57 | 35.25 | -4.68 | 48.95 | -18.38 |
| 3OPM_A | 30.41 | 36.59 | -6.18 | 45.97 | -15.56 |
| 3OPM_B | 34.98 | 38.79 | -3.81 | 46.93 | -11.95 |
| 3OPM_D | 33.26 | 38.38 | -5.12 | 51.33 | -18.07 |
| 3Q0T_B | 35.99 | 43.29 | -7.30 | 44.55 | -8.56 |
| 3QBJ_B | 51.3 | 64.62 | -13.32 | 69.3 | -18 |
| 3SWW_A | 29.44 | 34.97 | -5.53 | 35.83 | -6.39 |
| 3SWW_B | 28.87 | 35.45 | -6.58 | 35.76 | -6.89 |
| 3VJK_A (Teneligliptin) | 27.55 | 31.94 | -4.39 | 38.18 | -10.63 |
| 3VJK_B (Teneligliptin) | 27.68 | 32.01 | -4.33 | 36.25 | -8.57 |
| 3VJL_A | 17.04 | 26.76 | -9.72 | 26.13 | -9.09 |
| 3VJL_B | 17.1 | 27.25 | -10.15 | 24.35 | -7.25 |
| 3VJM_A | 15.87 | 30.89 | -15.02 | 28.37 | -12.5 |
| 3VJM_B | 21.83 | 30.96 | -9.13 | 27.43 | -5.6 |
| 3W2T_A (Vildagliptin) | 20.14 | 34.56 | -14.42 | 32.85 | -12.71 |
| 3W2T_B (Vildagliptin) | 18.25 | 34.51 | -16.26 | 31.21 | -12.96 |
| 4A5S_A | 14.88 | 33.37 | -18.49 | 21.55 | -6.67 |
| 4A5S_B | 19.16 | 34.90 | -15.74 | 25.49 | -6.33 |
| 4G1F_A | 9.84 | 23.77 | -13.93 | 42.76 | -32.92 |
| 4G1F_B | 18.92 | 22.71 | -3.79 | 40.03 | -21.11 |
| 4G1F_C | 24.07 | 24.35 | -0.28 | 47.23 | -23.16 |
| 4N8D_A | 16.35 | 38.41 | -22.06 | 23.49 | -7.14 |
| 4N8D_B | 19.55 | 40.69 | -21.14 | 27.96 | -8.41 |
| 4N8E_A | 18.8 | 25.39 | -6.59 | 23.18 | -4.38 |
| 4N8E_B | 4.23 | 26.45 | -22.22 | 26.8 | -22.57 |
| 4PNZ_A (Omarigliptin) | 19.31 | 38.16 | -18.85 | 26.65 | -7.34 |
| 4PNZ_B (Omarigliptin) | 20.17 | 38.52 | -18.35 | 27.25 | -7.08 |

* If the difference value is positive, the value is drawn in bold.
