# Supplementary material for: Comprehensive analysis of the Co-structures of dipeptidyl peptidase IV and its inhibitor
Source: BMC Struct Biol. 2016 Aug 5;16:11. doi: 10.1186/s12900-016-0062-8 (PMC4974693; doi:10.1186/s12900-016-0062-8)
Supplement: Additional file 7: — Table S3. B-factor of the second specific water O atom in a unit. (DOCX 41 kb) [file 12900_2016_62_MOESM7_ESM.docx]

**Table S3.** B-factor of the second specific water O atom in a unit.

| unit | B-factor of the second specific water O atom (1) | Average B-factor of all water O atoms in a unit (2) | difference  [(1)-(2)]* | Average B-factor of all heavy atoms of DPP-4 in a unit (3) | difference  [(1)-(3)]* |
| --- | --- | --- | --- | --- | --- |
| 1N1M_A | 26.19 | 30.47 | -4.28 | 31.43 | -5.24 |
| 1TKR_A | 7.37 | 22.49 | -15.12 | 24.44 | -17.07 |
| 1X70_A (Sitagliptin) | 19.49 | 33.13 | -13.64 | 26.34 | -6.85 |
| 1X70_B (Sitagliptin) | 22.94 | 33.92 | -10.98 | 27.13 | -4.19 |
| 2AJL_I | 13.87 | 14.52 | -0.65 | 22.01 | -8.14 |
| 2AJL_J | 4.71 | 14.97 | -10.26 | 20.55 | -15.84 |
| 2BUB_A | 10.61 | 29.06 | -18.45 | 38.71 | -28.1 |
| 2BUB_B | 27.37 | 28.82 | -1.45 | 38.77 | -11.4 |
| 2FJP_A | 24.35 | 32.38 | -8.03 | 33.03 | -8.68 |
| 2FJP_B | 24.46 | 32.70 | -8.24 | 33.42 | -8.96 |
| 2G5P_A | 27.22 | 42.94 | -15.72 | 41 | -13.78 |
| 2G5T_A | 19.82 | 37.12 | -17.30 | 30.32 | -10.5 |
| 2G63_B | 22.91 | 43.66 | -20.75 | 32.65 | -9.74 |
| 2HHA_A | 21.35 | 28.28 | -6.93 | 25.5 | -4.15 |
| 2HHA_B | 23.73 | 29.15 | -5.42 | 26.15 | -2.42 |
| 2I03_B | 39.37 | 51.70 | -12.33 | 47.83 | -8.46 |
| 2IIT_A | 20.76 | 31.54 | -10.78 | 29.55 | -8.79 |
| 2IIT_B | 18.01 | 32.02 | -14.01 | 30.31 | -12.3 |
| 2IIV_A | 17.96 | 29.28 | -11.32 | 26.26 | -8.3 |
| 2IIV_B | 17.86 | 30.28 | -12.42 | 26.96 | -9.1 |
| 2OAG_B | 23.36 | 41.26 | -17.90 | 37.51 | -14.15 |
| 2OGZ_A | 26.72 | 36.98 | -10.26 | 36.04 | -9.32 |
| 2OGZ_B | 28.36 | 37.02 | -8.66 | 34.71 | -6.35 |
| 2OLE_A | 33.1 | 30.54 | **2.56** | 31.32 | **1.78** |
| 2OLE_B | 25.04 | 28.04 | -3.00 | 30.35 | -5.31 |
| 2ONC_A | 40.37 | 42.70 | -2.33 | 48.85 | -8.48 |
| 2ONC_B | 29.81 | 45.03 | -15.22 | 51.49 | -21.68 |
| 2ONC_C | 34.76 | 45.00 | -10.24 | 51.5 | -16.74 |
| 2ONC_D | 55.73 | 48.73 | **7.00** | 64.63 | -8.9 |
| 2OPH_A | 19.8 | 27.92 | -8.12 | 25.49 | -5.69 |
| 2OPH_B | 20.77 | 28.82 | -8.05 | 25.93 | -5.16 |
| 2P8S_A | 20.53 | 31.89 | -11.36 | 27.18 | -6.65 |
| 2P8S_B | 25.98 | 32.34 | -6.36 | 28.25 | -2.27 |
| 2QJR_A | 53.99 | 54.11 | -0.12 | 56.73 | -2.74 |
| 2QJR_B | 57.8 | 52.24 | **5.56** | 56.7 | 1.1 |
| 2QOE_A | 20.87 | 27.61 | -6.74 | 28.39 | -7.52 |
| 2QOE_B | 22.38 | 28.02 | -5.64 | 28.51 | -6.13 |
| 2QT9_A | 15.45 | 29.00 | -13.55 | 23.17 | -7.72 |
| 2QT9_B | 13.79 | 29.60 | -15.81 | 23.88 | -10.09 |
| 2QTB_A | 18.47 | 28.84 | -10.37 | 26.23 | -7.76 |
| 2QTB_B | 19.74 | 29.52 | -9.78 | 26.25 | -6.51 |
| 2RIP_A | 31.81 | 22.75 | **9.06** | 38.3 | -6.49 |
| 3C43_A | 31.03 | 29.12 | **1.91** | 27.69 | **3.34** |
| 3C43_B | 32.7 | 29.98 | **2.72** | 28.14 | **4.56** |
| 3C45_A | 24.4 | 32.35 | -7.95 | 27.52 | -3.12 |
| 3C45_B | 26.99 | 33.00 | -6.01 | 28.13 | -1.14 |
| 3CCB_A | 35.26 | 39.72 | -4.46 | 50.4 | -15.14 |
| 3CCB_B | 33.85 | 41.60 | -7.75 | 49.38 | -15.53 |
| 3CCB_C | 38.11 | 41.49 | -3.38 | 50.98 | -12.87 |
| 3CCB_D | 40.18 | 39.45 | **0.73** | 52.78 | -12.6 |
| 3CCC_A | 32.06 | 43.28 | -11.22 | 53.52 | -21.46 |
| 3CCC_B | 32.11 | 40.73 | -8.62 | 51.6 | -19.49 |
| 3D4L_A | 22.47 | 36.39 | -13.92 | 31.39 | -8.92 |
| 3D4L_B | 25.78 | 37.32 | -11.54 | 32.32 | -6.54 |
| 3G0B_A (Alogliptin) | 40.56 | 39.53 | **1.03** | 51.76 | -11.2 |
| 3G0B_B (Alogliptin) | 38.59 | 41.91 | -3.32 | 51.34 | -12.75 |
| 3G0B_C (Alogliptin) | 40.61 | 43.32 | -2.71 | 53.58 | -12.97 |
| 3G0B_D (Alogliptin) | 41.24 | 40.73 | **0.51** | 52.24 | -11 |
| 3G0C_B | 54.65 | 42.11 | **12.54** | 47.36 | **7.29** |
| 3G0C_C | 54.72 | 42.28 | **12.44** | 48.21 | **6.51** |
| 3G0C_D | 54.94 | 44.59 | **10.35** | 49.4 | **5.54** |
| 3G0D_A | 36.16 | 47.49 | -11.33 | 51.69 | -15.53 |
| 3G0D_B | 34.13 | 49.11 | -14.98 | 51.74 | -17.61 |
| 3G0D_C | 39.5 | 48.65 | -9.15 | 52.24 | -12.74 |
| 3G0D_D | 38.31 | 51.88 | -13.57 | 56.83 | -18.52 |
| 3G0G_A | 40.74 | 41.55 | -0.81 | 46.44 | -5.7 |
| 3G0G_B | 48.66 | 43.86 | **4.80** | 47.61 | **1.05** |
| 3G0G_C | 36.09 | 43.70 | -7.61 | 51.08 | -14.99 |
| 3H0C_A | 21.68 | 33.03 | -11.35 | 35.92 | -14.24 |
| 3H0C_B | 28.51 | 33.36 | -4.85 | 40.49 | -11.98 |
| 3HAB_A | 20.29 | 29.09 | -8.80 | 25.88 | -5.59 |
| 3HAB_B | 18.55 | 29.94 | -11.39 | 25.97 | -7.42 |
| 3HAC_A | 23.55 | 36.27 | -12.72 | 34.66 | -11.11 |
| 3HAC_B | 25.29 | 36.31 | -11.02 | 34.68 | -9.39 |
| 3KWF_A | 30.64 | 36.56 | -5.92 | 40.46 | -9.82 |
| 3KWF_B | 27.82 | 36.43 | -8.61 | 40.1 | -12.28 |
| 3KWJ_B | 24.62 | 22.77 | **1.85** | 46.11 | -21.49 |
| 3NOX_A | 37.95 | 42.77 | -4.82 | 43.22 | -5.27 |
| 3O95_A | 37.73 | 30.84 | **6.89** | 49.04 | -11.31 |
| 3O95_B | 25.38 | 33.22 | -7.84 | 48.05 | -22.67 |
| 3O9V_B | 31.9 | 34.93 | -3.03 | 48.16 | -16.26 |
| 3O9V_C | 28.62 | 36.98 | -8.36 | 49.14 | -20.52 |
| 3O9V_D | 35.03 | 35.25 | -0.22 | 48.95 | -13.92 |
| 3OC0_A | 46.36 | 35.82 | **10.54** | 46.22 | **0.14** |
| 3OPM_A | 32.74 | 36.59 | -3.85 | 45.97 | -13.23 |
| 3OPM_B | 33.42 | 38.79 | -5.37 | 46.93 | -13.51 |
| 3OPM_C | 47.63 | 41.21 | **6.42** | 63.8 | -16.17 |
| 3OPM_D | 39.83 | 38.38 | **1.45** | 51.33 | -11.5 |
| 3Q0T_A | 31.31 | 41.89 | -10.58 | 43.39 | -12.08 |
| 3QBJ_B | 62.55 | 64.62 | -2.07 | 69.3 | -6.75 |
| 3SWW_A | 26.61 | 34.97 | -8.36 | 35.83 | -9.22 |
| 3SWW_B | 28.73 | 35.45 | -6.72 | 35.76 | -7.03 |
| 3VJK_A (Teneligliptin) | 26.93 | 31.94 | -5.01 | 38.18 | -11.25 |
| 3VJK_B (Teneligliptin) | 18.68 | 32.01 | -13.33 | 36.25 | -17.57 |
| 3VJL_A | 32.63 | 26.76 | **5.87** | 26.13 | **6.5** |
| 3VJL_B | 22.72 | 27.25 | -4.53 | 24.35 | -1.63 |
| 3VJM_A | 27.89 | 30.89 | -3.00 | 28.37 | -0.48 |
| 3VJM_B | 28.23 | 30.96 | -2.73 | 27.43 | **0.8** |
| 3W2T_A (Vildagliptin) | 28.38 | 34.56 | -6.18 | 32.85 | -4.47 |
| 3W2T_B (Vildagliptin) | 23.73 | 34.51 | -10.78 | 31.21 | -7.48 |
| 4A5S_A | 18.28 | 33.37 | -15.09 | 21.55 | -3.27 |
| 4A5S_B | 24.12 | 34.90 | -10.78 | 25.49 | -1.37 |
| 4N8D_A | 18.02 | 38.41 | -20.39 | 23.49 | -5.47 |
| 4N8D_B | 22.67 | 40.69 | -18.02 | 27.96 | -5.29 |
| 4N8E_A | 25.55 | 25.39 | **0.16** | 23.18 | **2.37** |
| 4N8E_B | 15.43 | 26.45 | -11.02 | 26.8 | -11.37 |
| 4PNZ_A (Omarigliptin) | 19.82 | 38.16 | -18.34 | 26.65 | -6.83 |
| 4PNZ_B (Omarigliptin) | 20.81 | 38.52 | -17.71 | 27.25 | -6.44 |

* If the difference value is positive, the value is drawn in bold.
