# Supplementary material for: Comprehensive analysis of the Co-structures of dipeptidyl peptidase IV and its inhibitor
Source: BMC Struct Biol. 2016 Aug 5;16:11. doi: 10.1186/s12900-016-0062-8 (PMC4974693; doi:10.1186/s12900-016-0062-8)
Supplement: Additional file 8: — Table S4. DPP-4 inhibitory activity. (DOCX 30 kb) [file 12900_2016_62_MOESM8_ESM.docx]

**Table S4.** DPP-4 inhibitory activity.

| PDB ID | IC_50_ / nM | Ki / nM | Web Site*/Reference |
| --- | --- | --- | --- |
| 1N1M | 3000 | 2000 | BindingDB |
| 1RWQ | 0.1 |  | BindingDB |
| 1TKR | unknown |  |  |
| 1X70 (Sitagliptin) | 18 |  | BindingDB |
| 2AJL |  | 13 | PDBbind |
| 2BUB | 90 |  | BindingDB |
| 2FJP | 4 | 4.3 | BindingDB |
| 2G5P |  | 3.8 | BindingDB |
| 2G5T |  | 0.82 | BindingDB |
| 2G63 |  | 6.7 | BindingDB |
| 2HHA | 122 |  | BindingDB |
| 2I03 |  | 1 | BindingDB |
| 2I78 |  | 1.3 | BindingDB |
| 2IIT | 2.6 |  | BindingDB |
| 2IIV | 6.6 |  | BindingDB |
| 2JID |  | 400 | PDBbind |
| 2OAG |  | 3.4 | BindingDB |
| 2OGZ | 84 |  | BindingDB |
| 2OLE | 70 | 56.2 | BindingDB |
| 2ONC | 13 |  | BindingDB |
| 2OPH | 16 |  | BindingDB |
| 2OQI |  | 3.2 | BindingDB |
| 2OQV |  | 4 | BindingDB |
| 2P8S | 21 |  | BindingDB |
| 2QJR | 6.4 |  | BindingDB |
| 2QOE | 25 |  | BindingDB |
| 2QT9 | 2.3 |  | BindingDB |
| 2QTB | 4.8 |  | BindingDB |
| 2RGU (Linagliptin) | 1 |  | BindingDB |
| 2RIP | 65 |  | PDBbind |
| 3BJM (Saxagliptin) |  | 0.6 | BindingDB |
| 3C43 | 19 |  | PDBbind |
| 3C45 | 0.21 |  | BindingDB |
| 3CCB | 30000 |  | BindingDB |
| 3CCC | 8 |  | BindingDB |
| 3D4L | 32 |  | BindingDB |
| 3EIO | 223 |  | BindingDB |
| 3F8S | 11 |  | BindingDB |
| 3G0B (Alogliptin) | 7 |  | BindingDB |
| 3G0C | 2000 |  | PDBbind |
| 3G0D | 5 | 2 | BindingDB/PDBbind |
| 3G0G | 5 |  | BindingDB |
| 3H0C | 0.38 |  | BindingDB |
| 3HAB | 4.2 |  | BindingDB |
| 3HAC | 8.3 |  | BindingDB |
| 3KWF | 6.8 |  | BindingDB |
| 3KWJ | 0.5 |  | BindingDB |
| 3NOX |  | 2.2 | BindingDB |
| 3O95 | 5.3 |  | BindingDB |
| 3O9V | 18 |  | BindingDB |
| 3OC0 | 520 |  | BindingDB |
| 3OPM | 47 |  | BindingDB |
| 3Q0T | 2.7 |  | BindingDB |
| 3QBJ | unknown |  |  |
| 3SWW |  | 4 | BindingDB |
| 3SX4 |  | 3 | BindingDB |
| 3VJK (Teneligliptin) | 0.37 |  | BindingDB |
| 3VJL | 5.6 |  | BindingDB |
| 3VJM | 0.37 |  | BindingDB |
| 3W2T (Vildagliptin) | 3.5 |  | BindingDB |
| 3WQH (Anagliptin) | 3.8 |  | BindingDB |
| 4A5S | 17 |  | BindingDB |
| 4G1F | 17 |  | BindingDB |
| 4JH0 |  | 0.94 | PDBbind |
| 4LKO |  | 6 | PDBbind |
| 4N8D | 11900 |  | PDBbind |
| 4N8E | 20 |  | PDBbind |
| 4PNZ (Omarigliptin) | 1.6 | 0.8 | [22] |
| (Trelagliptin) | 4 |  | [15] |

*IC_50_ and Ki was quoted from the website “The binding data base”, [http://www.bindingdb.org/bind/index.jsp [24](http://www.bindingdb.org/bind/index.jsp%20%5b24)] or the website “PDB bind”, http://www.pdbbind-cn.org/index.asp [25].
